# The Tumour Suppressor TMEM127 Is a Nedd4-Family E3 Ligase Adaptor Required by *Salmonella* SteD to Ubiquitinate and Degrade MHC Class II Molecules

**DOI:** 10.1016/j.chom.2020.04.024

**Published:** 2020-07-08

**Authors:** Eric Alix, Camilla Godlee, Ondrej Cerny, Samkeliso Blundell, Romina Tocci, Sophie Matthews, Mei Liu, Jonathan N. Pruneda, Kirby N. Swatek, David Komander, Tabitha Sleap, David W. Holden

**Affiliations:** 1 MRC Centre for Molecular Bacteriology and Infection, Imperial College London, Armstrong Road, London SW7 2AZ, UK; 2 Department of Molecular Microbiology and Immunology, Oregon Health & Science University, 3181 S.W. Sam Jackson Park Road, Portland, OR 97239, USA; 3 Ubiquitin Signalling Division, The Walter and Eliza Hall Institute of Medical Research, 1G Royale Parade, 3052 Parkville, Melbourne, Australia; 4 Department of Molecular Machines and Signaling, Max Planck Institute of Biochemistry, Martinsried, Germany

**Keywords:** *Salmonella*, SteD, MHCII, CRISPR screen, WWP2, TMEM127, ubiquitination, lysosomal degradation, dendritic cells

## Abstract

The *Salmonella enterica* effector SteD depletes mature MHC class II (mMHCII) molecules from the surface of infected antigen-presenting cells through ubiquitination of the cytoplasmic tail of the mMHCII β chain. Here, through a genome-wide mutant screen of human antigen-presenting cells, we show that the NEDD4 family HECT E3 ubiquitin ligase WWP2 and a tumor-suppressing transmembrane protein of unknown biochemical function, TMEM127, are required for SteD-dependent ubiquitination of mMHCII. Although evidently not involved in normal regulation of mMHCII, TMEM127 was essential for SteD to suppress both mMHCII antigen presentation in mouse dendritic cells and MHCII-dependent CD4^+^ T cell activation. We found that TMEM127 contains a canonical PPxY motif, which was required for binding to WWP2. SteD bound to TMEM127 and enabled TMEM127 to interact with and induce ubiquitination of mature MHCII. Furthermore, SteD also underwent TMEM127- and WWP2-dependent ubiquitination, which both contributed to its degradation and augmented its activity on mMHCII.

## Highlights

• TMEM127 and WWP2 are required for MHCII depletion by *Salmonella* SteD
• Tumor suppressor TMEM127 is an adaptor for oncoprotein E3 ligase WWP2
• SteD interacts with TMEM127 to induce ubiquitination and degradation of mature MHCII
• SteD also undergoes TMEM127- and WWP2-dependent ubiquitination

## Graphical Abstract

**Figure:**
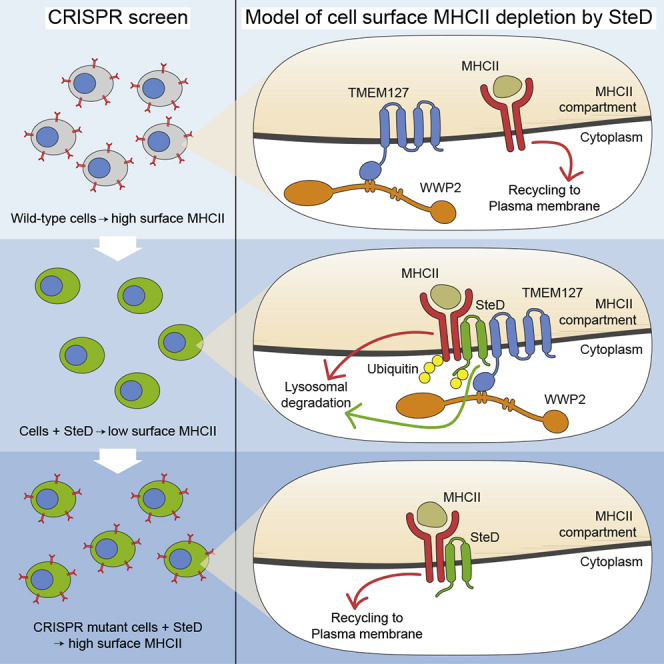

## Teaser

*Salmonella* inhibits the adaptive immune response by reducing cell surface levels of mature MHC class II. Alix et al. reveal that the *Salmonella* effector SteD co-opts TMEM127, a tumor suppressor protein of previously unknown function. TMEM127 binds the E3 ubiquitin ligase WWP2 enabling ubiquitination and degradation of MHCII and SteD.

## Introduction

The ability of *Salmonella enterica* to cause life-threatening diseases such as typhoid fever requires many bacterial virulence proteins (effectors) that interfere with both innate and adaptive immune responses, both of which are normally involved in the control and elimination of the pathogen. Innate responses are countered by *Salmonella* effectors that are translocated into infected host cells (including epithelial cells, macrophages, and dendritic cells [DCs]) by the *Salmonella* pathogenicity island (SPI)-1 and SPI-2 type III secretion systems (T3SS). These effectors are frequently enzymes that catalyze post-translational modification of host innate signalling pathway proteins (Jennings et al., 2017).

CD4^+^ T cells are the major component of the adaptive immune system involved in elimination of *S. enterica* from systemic tissues of both mice (Kupz et al., 2014) and humans (Dunstan et al., 2014). CD4^+^ T cells are activated by surface major histocompatibility complex class II molecules (MHCII) of antigen presenting cells, such as DCs. DCs internalise *Salmonella* cells at the gut epithelium and transport them to mesenteric lymph nodes, where T cell responses are initiated (Cerny and Holden, 2019).

In DCs, the amount of peptide-loaded, mature major histocompatibility complex class II (mMHCII) at the cell surface reflects the rates of both endocytosis and recycling from MHCII-containing endosomes (known as MHCII or antigen processing compartments). In immature DCs, surface mMHCII is limited by the membrane-associated RING-CH (MARCH)1 E3 ubiquitin ligase, which targets molecules present in MHCII compartments and ubiquitinates the cytoplasmic tail of the MHCII β chain. This enables recognition by the endosomal sorting required for transport (ESCRT) complex, internalization of mMHCII into intra-luminal vesicles, and its endo-lysosomal degradation (Roche and Furuta, 2015). Upon DC activation, MARCH1 expression ceases, allowing non-ubiquitinated mMHCII to recycle to the plasma membrane to initiate CD4^+^ T cell responses (Cho et al., 2015). *Salmonella* depletes mMHCII but not immature (invariant chain-bound) MHCII from the plasma membrane of infected DCs and decreases the ability of DCs to activate T cells (Cheminay et al., 2005, Lapaque et al., 2009, Tobar et al., 2006). We showed previously that the highly conserved SPI-2 T3SS effector SteD (Jennings et al., 2017) is required and sufficient for this process and used the MHCII-expressing Mel Juso cell line to implicate the MARCH1 homologue, MARCH8, in SteD-dependent ubiquitination of the DRβ chain of mMHCII (Bayer-Santos et al., 2016).

To gain further insights into the mechanism by which SteD depletes surface mMHCII we undertook both targeted and unbiased genetic approaches. Targeted knockouts of MARCH8 in Mel Juso cells and MARCH1 in dendritic cells failed to support a role of these enzymes in SteD function. Instead, a genome-wide mutant screen led to the identification of two proteins—the NEDD4 family homologous to E6AP C terminus (HECT) E3 ubiquitin ligase WWP2 and the transmembrane protein TMEM127—that are required for *Salmonella* Typhimurium (hereafter referred to as *Salmonella*) SteD-dependent ubiquitination of mMHCII. Although having no detectable influence on the normal regulation of MHCII, we found that TMEM127 is essential for SteD to suppress both mMHCII antigen presentation in dendritic cells and MHCII-dependent CD4^+^ T cell activation. TMEM127 contains a canonical PPxY motif that is required for binding to WWP2. SteD binds to TMEM127 and enables TMEM127 to ubiquitinate mMHCII through WWP2. Furthermore, SteD also undergoes TMEM127- and WWP2-dependent ubiquitination, which both contributes to its own degradation and augments its activity on mMHCII.

## Results

### A Genome-Wide Mutant Screen to Identify Genes Required for SteD-Dependent Depletion of Surface mMHCII

RNA knockdown experiments in human melanoma-derived Mel Juso cells (extensively used for studying MHCII trafficking) suggested that the E3 ligase MARCH8 is required for SteD-induced mMHCII ubiquitination (Bayer-Santos et al., 2016). In view of incomplete inactivation of MARCH8 and the possibility of off-target effects, we generated *MARCH8*^*−/−*^ Mel Juso cells by CRISPR-Cas9 mutagenesis (Figure S1A) and infected them with wild-type *Salmonella* or *steD* mutant bacteria. As previously reported (Bayer-Santos et al., 2016), SteD was required for a dramatic reduction in cell surface mMHCII (Figure S1B), as measured by flow cytometry using mAb L243, which specifically recognizes mature HLA-DR. However, the same effect was also observed in the absence of MARCH8 (Figure S1B). Tetra- and penta-ubiquitinated MHCII in uninfected and infected Mel Juso cells was much reduced in the absence of MARCH8 (Figure S1C), in agreement with previous work on the endogenous regulation of mMHCII (Lapaque et al., 2009, Ma et al., 2012). In contrast, SteD clearly increased the amounts of di-ubiquitinated mMHCII, and MARCH8 was not required for this (Figure S1C). We confirmed that surface levels of MHCII in a *March1*^*−/−*^ mouse-derived MutuDC cell line were higher than in wild-type cells (Wilson et al., 2018) (Figure S1D). In mouse cells, it is not possible to discriminate between immature and mature MHCII molecules, so measurements of MHCII in these cells reflect both forms. However, SteD-dependent depletion of total surface MHCII by intracellular *Salmonella* was similar in wild-type or *March1*^*−/−*^ cells (Figure S1E). Together, these results establish that neither MARCH8 nor MARCH1 are involved in SteD function and suggest that our previous results (Bayer-Santos et al., 2016) were due to off-target effects or other artifacts.

**Figure 1:**
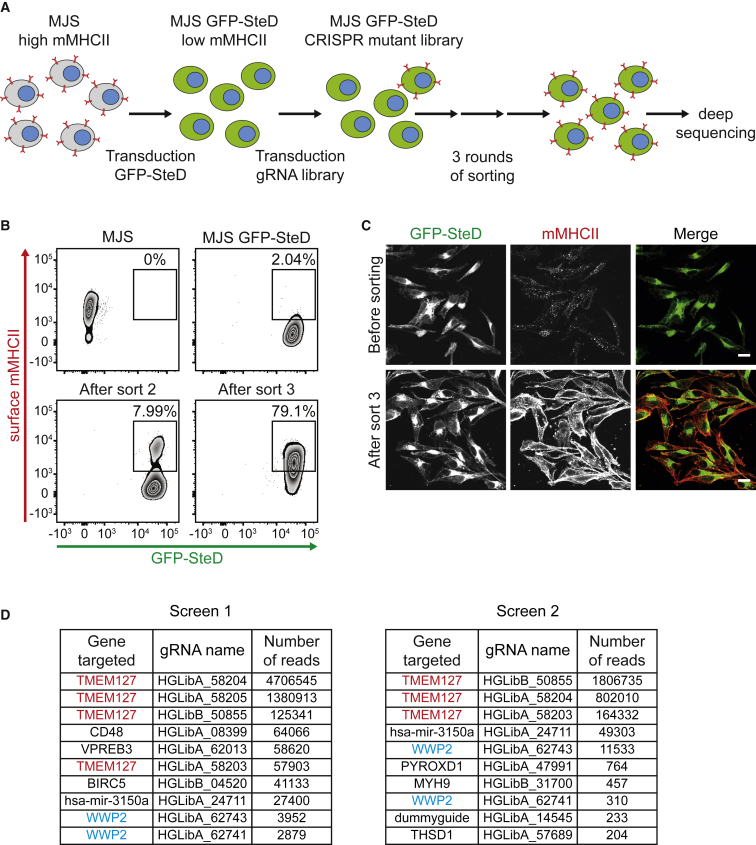
Genome-Wide Mutant Screen Identifies Two Proteins Required for SteD-Dependent Depletion of Surface mMHCII (A) Schematic of genome-wide CRISPR/Cas9 mutant screen of Mel Juso cells (MJS) to identify genes required for SteD-dependent depletion of surface mMHCII. (B) FACS plots representative of the CRISPR/Cas9 screen, revealing enrichment of GFP^high^/L243^high^ cells after mutagenesis and three rounds of sorting. (C) Confocal immunofluorescence microscopy of GFP-SteD (green) and surface mMHCII (red) before sorting and after three rounds of enrichment. Scale bar, 10 μm. (D) gRNAs in enriched cells were subjected to deep sequencing. The 10 most enriched gRNAs, their corresponding genes and number of sequencing reads from two screens are shown. Data taken from Table S1. See also Figure S1; Table S1.

To identify host molecules required by SteD to deplete mMHCII from the host cell plasma membrane we carried out two CRISPR/Cas9-based genome-wide mutant screens (Joung et al., 2017) in Mel Juso cells (Figure 1A). Mel Juso cells stably expressing Cas9 and GFP-SteD (MJS GFP-SteD cells) had a much lower level of surface mMHCII compared to Cas9-expressing wild-type cells (Figure 1B). We generated two pooled libraries of mutants in MJS GFP-SteD cells using the Geckov2 gRNA library (Sanjana et al., 2014) (6 gRNA/ORF for 20,000 ORFs of the human genome and 4 gRNAs/miRNA). Mutants resulting in high mMHCII surface levels were enriched by three successive rounds of cell sorting by flow cytometry (Figure 1B). Immunofluorescence microscopy confirmed strong surface labelling of mMHCII following enrichment, despite normal production and localization of GFP-SteD (Figure 1C). The gRNAs in these cells were subjected to PCR amplification and deep sequencing (Table S1); the gRNAs representing the 10 most frequent hits for the two screens were ranked in order of abundance of read count per gRNA (Figure 1D). Of these, four gRNAs targeting *TMEM127* accounted for the three most abundant sequence reads in each screen (Figure 1D). Overall, gRNAs targeting *TMEM127* represented 97% of the total gRNAs sequenced. Only one other gene (*WWP2*) was represented by two gRNAs among the top ten hits in each screen (Figure 1D). One gRNA (24711) targeting the microRNA mir-3150 was also enriched in each screen. However, three other gRNAs present in the library targeting mir-3150 were completely absent from sequencing reads.

### TMEM127 and WWP2 Are Required for SteD-Dependent mMHCII Surface Level Decrease and Ubiquitination

**Figure 2:**
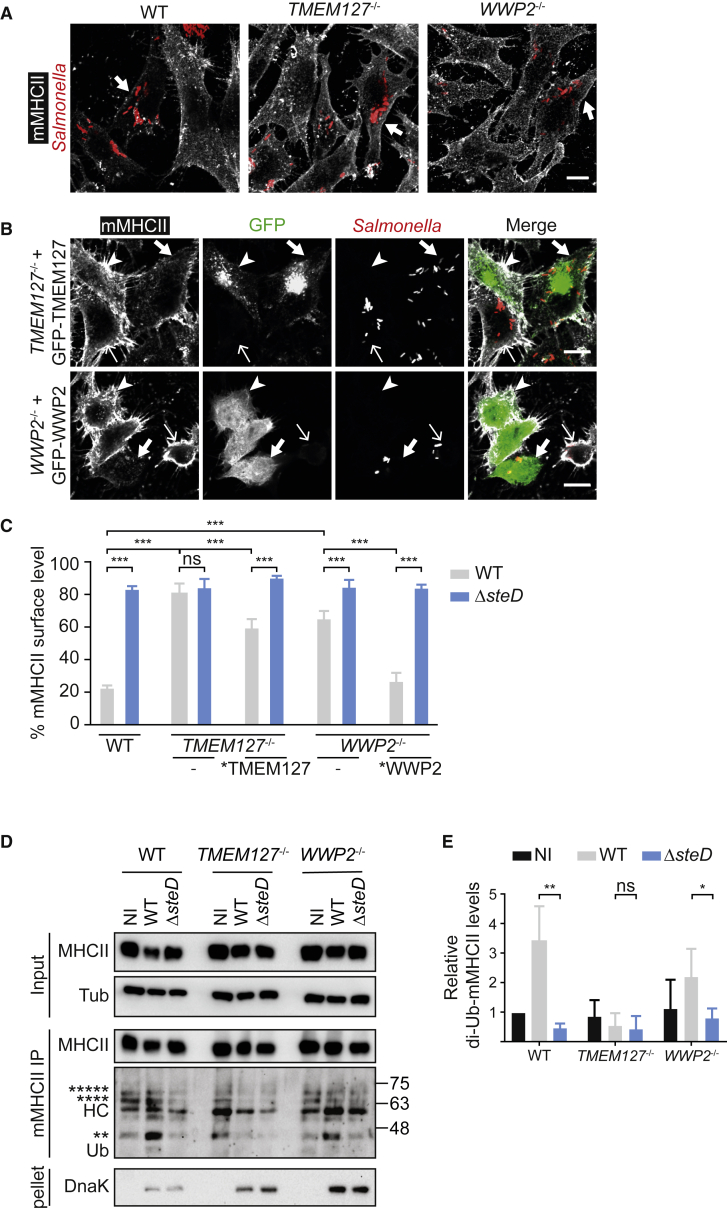
Validation of Mutant Screen Hits (A) TMEM127 and WWP2 are required for reduction of surface mMHCII by *Salmonella*. Representative confocal immunofluorescence microscopy images of wild-type (WT), *TMEM127*^*−/−*^, or *WWP2*^*−/−*^ Mel Juso cells infected with mCherry-expressing WT *Salmonella* (red). Cells were fixed 20 h p.i. and labelled for surface mMHCII (L243 antibody, white). Arrows indicate surface mMHCII of infected cells. Scale bar, 10 μm. (B) Mutant complementation. Representative confocal immunofluorescence microscopy images of *TMEM127*^*−/−*^ or *WWP2*^*−/−*^ Mel Juso cells expressing GFP-TMEM127 or GFP-WWP2, respectively (green), then infected with mCherry-expressing wild-type *Salmonella* (red). Cells were fixed 20 h p.i. and labelled for surface mMHCII (L243 antibody, white). Thick arrows show transfected, infected cells. Narrow arrows show non-transfected, infected cells. Arrowheads show transfected, uninfected cells. Scale bar, 10 μm. (C) Quantification of mMHCII surface levels in mutant and complemented Mel Juso cells infected with WT or *steD* mutant *Salmonella*. Cells were analysed by flow cytometry and amounts of surface mMHCII in infected cells are expressed as a percentage of uninfected cells in the same sample. ^∗^TMEM127 and ^∗^WWP2 correspond to GFP-tagged proteins. Data are from 3 independent experiments and show means ± SD. ^∗∗∗^ p < 0.001, ns, not significant (two-way ANOVA followed by Tukey’s multiple comparison test). (D) WT, *TMEM127*^*−/−*^, or *WWP2*^*−/−*^ Mel Juso cells infected with WT or *steD* mutant *Salmonella* were lysed and proteins were immunoprecipitated with L243 antibody. Samples were analysed by immunoblot using anti-DRα (MHCII) anti-tubulin (Tub), anti-ubiquitin (Ub), and anti-DnaK (as a *Salmonella* marker) antibodies. HC – IgG heavy chain. Ubiquitin blot detects ubiquitinated mMHCII β chain (unmodified β chain –29 KDa). Bands with masses corresponding to di-, tetra-, and penta-ubiquitinated mMHCII β chain are indicated by ^∗∗^, ^∗∗∗∗^, and ^∗∗∗∗∗^, respectively. Protein size markers (kDa) are indicated on right. (E) Quantification of intensity of di-ubiquitinated mMHCII signal represented in (D). Data are from 5 independent experiments and show means ± SD. ^∗∗^ p < 0.01, ^∗^ p < 0.05, ns, not significant (Student’s t test). See also Figure S2.

Mutations in TMEM127 confer susceptibility to pheochromocytoma, a tumour of the adrenal gland (Qin et al., 2010). It is a 238-amino acid transmembrane protein of unknown biochemical function, involved in mammalian target of rapamycin (mTOR) nutrient sensing (Deng et al., 2018) and insulin signalling (Srikantan et al., 2019). WWP2 belongs to the NEDD4 family of HECT E3 ubiquitin ligases (Scheffner and Kumar, 2014). NEDD4 ligases control ubiquitination and regulation of many proteins including membrane proteins (Foot et al., 2008). We generated independent CRISPR-Cas9 knockouts of *WWP2* and *TMEM127* in Mel Juso cells (Figures S2A and S2B). There were no detectable differences in mMHCII surface levels between wild-type and knockout cells in the absence of infection (Figure S2C). However, the ability of wild-type *Salmonella* to induce SteD-dependent depletion of surface mMHCII in infected cells was strongly impaired in cells lacking TMEM127 or WWP2 (Figures 2A–2C). Whereas absence of TMEM127 abolished all of the SteD effect, the WWP2 knockout retained residual activity (Figure 2C). Ectopic expression of GFP-TMEM127 or GFP-WWP2 in knockout cells complemented the corresponding mutations partially and completely, respectively (Figures 2B, 2C, and S3A). The effects in knockout cells were accompanied by a significant reduction in SteD-induced di-ubiquitination of mMHCII (Figures 2D and 2E). A catalytic-dead point mutant of WWP2 (GFP-WWP2[C/A]) (Mund et al., 2015) failed to complement *WWP2* knockout cells (Figure S3B), showing that SteD requires WWP2’s catalytic activity to function.

### SteD Requires Tmem127 to Deplete Surface MHCII in Dendritic Cells and to Inhibit T Cell Proliferation

**Figure 3:**
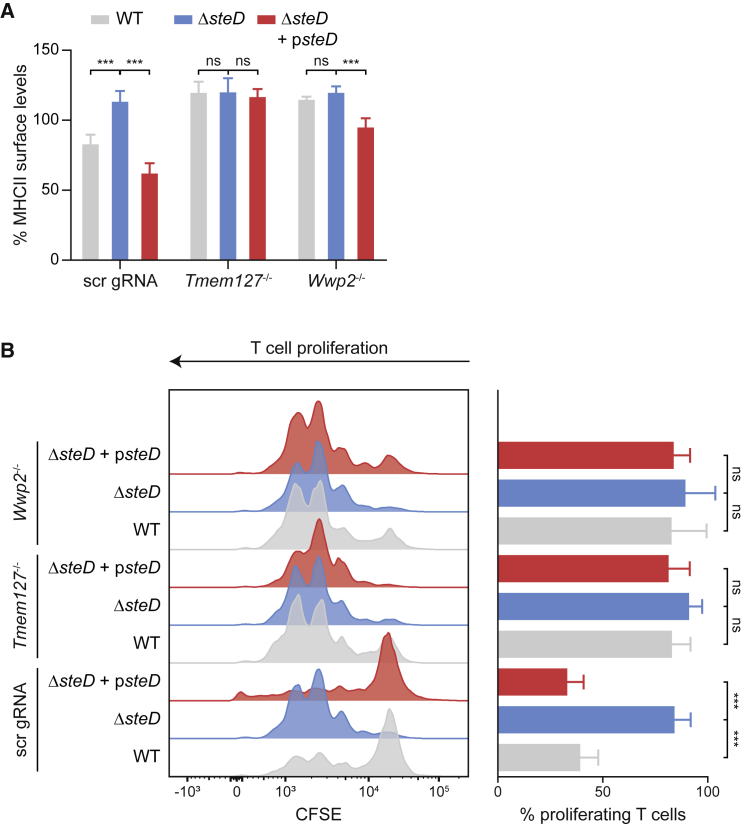
Tmem127 and Wwp2 Are Important for Function of SteD in Dendritic Cells (A) Quantification of total MHCII surface levels in scrambled (scr) gRNA, *Tmem127*^*−/−*^ or *Wwp2*^*−/−*^ MutuDCs, infected with the indicated *Salmonella* strains. Cells were analysed by flow cytometry 20 h p.i., and amounts of surface MHCII in infected cells are expressed as a percentage of uninfected cells in the same sample. Data are from 3 independent experiments and show means ± SD. ^∗∗∗^ p < 0.001, ns, not significant (two-way ANOVA followed by Tukey’s multiple comparison test). (B) Representative FACS histograms (left) showing OVA-dependent T cell proliferation measured by CFSE dilution in different conditions after incubation with scrambled (scr) gRNA, *Tmem127*^*−/−*^ or *Wwp2*^*−/−*^ MutuDCs infected with the indicated *Salmonella* strains and exposed to OVA peptide. Quantification of percentage proliferating T cells for each condition (right) representing 3 independent experiments and showing means ± SD. ^∗∗∗^ p < 0.001, ns, not significant (two-way ANOVA followed by Tukey’s multiple comparison test). See also Figure S2.

To investigate the involvement of Wwp2 and Tmem127 in SteD-dependent depletion of total surface MHCII in DCs, we generated gene knockouts in the mouse MutuDC cell line by CRISPR-Cas9 mutagenesis (Figures S2D and S2E). Activation of wild-type MutuDCs by infection with *steD* mutant *Salmonella* led to significantly higher surface MHCII levels than DCs that were not exposed to bacteria (Figure S2F), and these levels were unaffected by the absence of either Wwp2 or Tmem127 (Figure S2F). In wild-type *Salmonella*-infected MutuDCs that had been treated with scrambled gRNAs, there was a clear SteD-dependent reduction of surface MHCII (Figure 3A), in agreement with previous results using primary DCs (Bayer-Santos et al., 2016). Consistent with results obtained with Mel Juso cells (Figure 2C), the effect of SteD was completely abrogated in *Tmem127*^*−/−*^ MutuDCs and partially in *Wwp2*^*−/−*^ MutuDCs (Figure 3A). We previously reported a strong SteD-dependent inhibition of CD4^+^ T cell proliferation by *Salmonella*-infected DCs (Bayer-Santos et al., 2016). Loss of Tmem127 in MutuDCs had no effect on Ovalbumin (OVA)-dependent CD4^+^ T cell proliferation induced by MutuDCs infected with *steD* mutant *Salmonella* and pulsed with OVA peptide (Figure 3B). However, wild-type DCs infected with *Salmonella* expressing SteD strongly inhibited T cell proliferation and this was dependent on Tmem127 and Wwp2 (Figure 3B). We conclude from these results that Tmem127 and Wwp2 are not involved in the normal process of MHCII-mediated antigen presentation but are important for the function of SteD in suppressing surface MHCII in infected DCs and for MHCII-dependent CD4^+^ T cell proliferation.

### TMEM127 Is a NEDD4 Family-Interacting Protein

**Figure 4:**
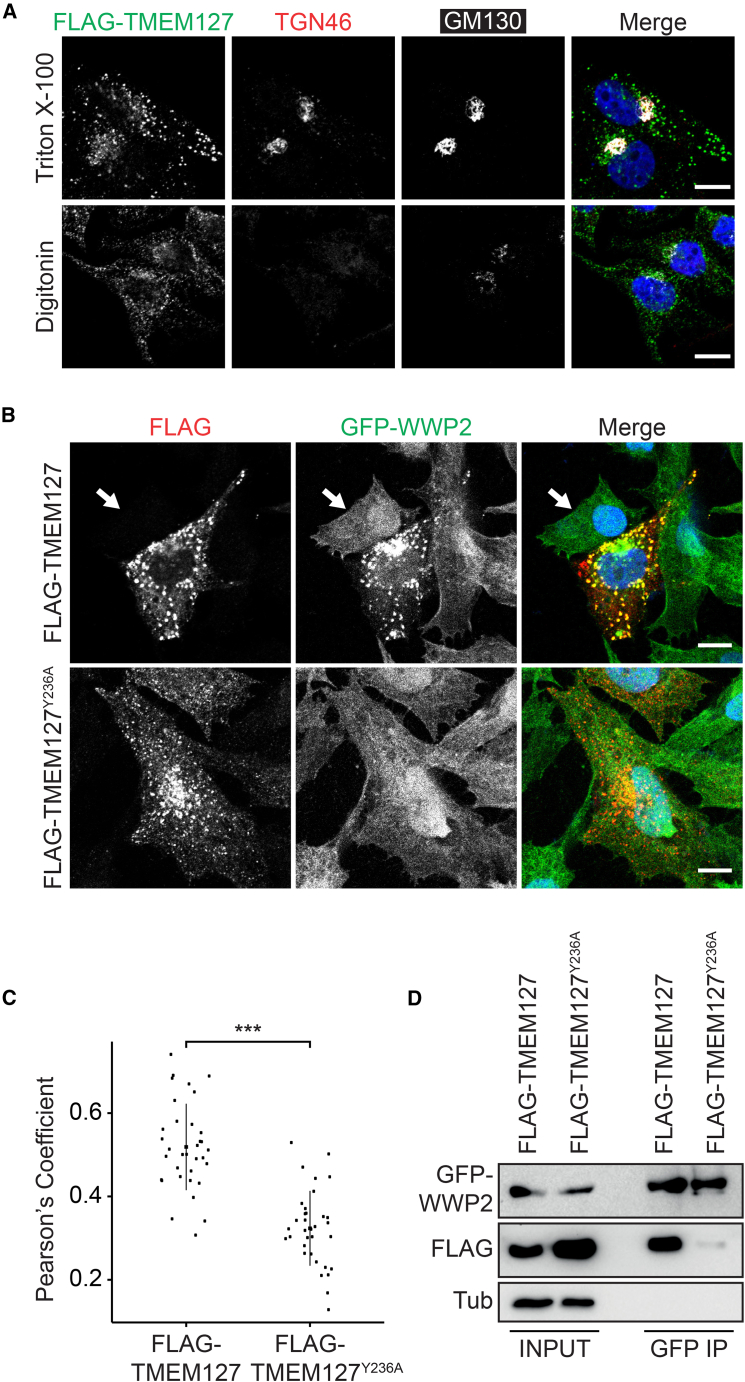
TMEM127 Is a NEDD4 Family-Interacting Protein (A) Representative confocal immunofluorescence microscopy images of Mel Juso cells expressing FLAG-TMEM127 (green). Cells were completely or semi-permeabilized with Triton X-100 (top) or digitonin (bottom) to discriminate between luminal and cytoplasmic epitopes, respectively. Antibodies recognizing the luminal portion of TGN46 or cytoplasmic GM130 were used as controls. Scale bar, 10 μm. (B) Representative confocal immunofluorescence microscopy images of *TMEM127*^*−/−*^ Mel Juso cells expressing GFP-WWP2 (green) and FLAG-TMEM127 or FLAG-TMEM127^Y236A^. Cells were fixed and labelled for FLAG-TMEM127 (anti-FLAG antibody, red) and DAPI (blue). Representative image shows relocalization and colocalisation of GFP-WWP2 in cells expressing FLAG-TMEM127 but not in cells expressing FLAG-TMEM127^Y236A^. Arrow indicates diffuse cytoplasmic signal of GFP-WWP2 in a cell not expressing FLAG-TMEM127 or FLAG-TMEM127^Y236A^. Scale bar, 10 μm. (C) Pearson’s correlation coefficients for colocalization between GFP-WWP2 and FLAG-TMEM127 or FLAG-TMEM127^Y236A^. Data are representative of three independent experiments. Each dot represents the value for one cell. Error bars show mean ± SD. ^∗∗∗^ p < 0.001 (Student’s t test). (D) Coimmunoprecipitation of FLAG-TMEM127 but not FLAG-TMEM127^Y236A^ with GFP-WWP2. HEK293ET cells expressing GFP-WWP2 and either FLAG-TMEM127 or FLAG-TMEM127^Y236A^ were lysed and proteins were immunoprecipitated with GFP-trap beads. Samples were analysed by immunoblot using anti-GFP, anti-FLAG, and anti-tubulin (Tub) antibodies. Representative of 3 independent experiments.

NEDD4 ubiquitin ligases including WWP2 contain an N-terminal C2 lipid-binding domain, several WW domains and a C-terminal catalytic HECT region (Zou et al., 2015). WW domains recognize a consensus PPxY sequence in substrates or cognate adaptors, including the NEDD4 family-interacting proteins (NDFIPs) NDFIP1 and NDFIP2 (Shearwin-Whyatt et al., 2006). Inspection of the amino acid sequences of SteD, MHCII molecules, and TMEM127 revealed that TMEM127 alone possesses a PPAY sequence, at its C-terminal extremity (Figure S2A). TMEM127 is predicted to have three (Qin et al., 2010) or possibly four (Tsirigos et al., 2015) transmembrane regions. To determine the topology of TMEM127, and for other biochemical studies, we used a FLAG-tagged version (Deng et al., 2018). As expected, FLAG-TMEM127 (shown to be functional by complementation of surface mMHCII depletion by SteD [Figures S4A and S4B]) had a punctate distribution in Mel Juso cells (Figure 4A), reflecting its presence in endosomal compartments (Qin et al., 2014). Semi-permeabilization and antibody-labelling of these cells showed that the N-terminal domain of TMEM127 is exposed to the cytoplasm (Figure 4A). We hypothesized that TMEM127 binds WWP2 through its PPAY sequence. GFP-WWP2 was located diffusely in the cytosol of Mel Juso cells (Figure 4B). Co-expression of both proteins caused a dramatic relocalisation of GFP-WWP2 to FLAG-TMEM127-positive vesicles (Figure 4B). This alteration was abrogated by substitution of Y236 of the PPAY sequence of TMEM127 (Figures 4B and 4C), indicating that the C-terminal region of TMEM127 faces the cytoplasm, and that the protein therefore has four transmembrane domains. In HEK293ET cells (which do not express MHCII) GFP-WWP2 and FLAG-TMEM127 interacted, and this was largely dependent on Y236 of TMEM127 (Figure 4D). Since TMEM127 does not display sequence similarity to NDFIP1 or NDFIP2, we conclude that TMEM127 is a distinct type of NDFIP that requires its PPAY sequence to interact with WWP2.

### SteD Binds to TMEM127 and Promotes TMEM127-mMHCII Interactions

**Figure 5:**
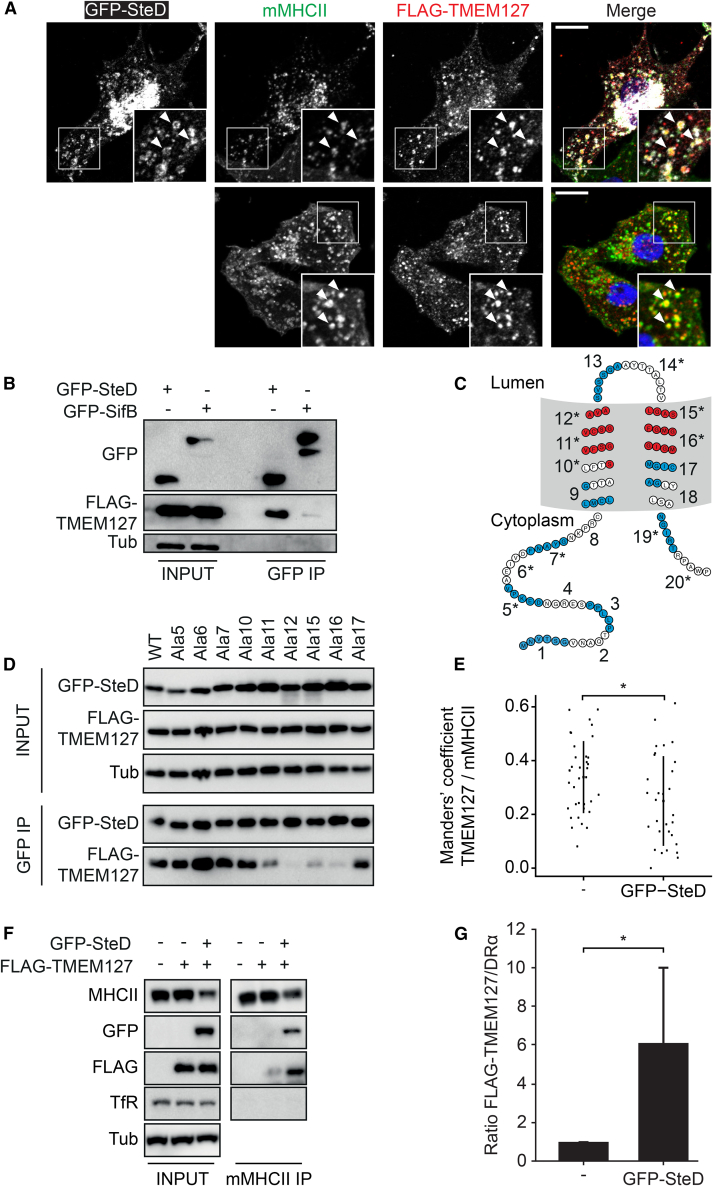
SteD Binds to TMEM127 and Enables Interaction between TMEM127 and mMHCII (A) Representative confocal immunofluorescence microscopy images of Mel Juso cells expressing FLAG-TMEM127 and GFP-SteD (white; upper panel) or only FLAG-TMEM127 (lower panel). Cells were fixed and labelled for total mMHCII (L243 antibody, green), FLAG-TMEM127 (anti-FLAG, red), and DNA (DAPI). Magnified boxed area shows vesicular colocalization of the three proteins (arrowheads). Scale bar, 10 μm. (B) Coimmunoprecipitation of FLAG-TMEM127 with GFP-SteD but not GFP-SifB. HEK293ET cells expressing FLAG-TMEM127 and GFP-SteD or GFP-SifB were lysed and proteins were immunoprecipitated with GFP-trap beads. Samples were analyzed by immunoblot using anti-GFP, anti-FLAG, and anti-tubulin (Tub) antibodies. Representative of 3 independent experiments. (C) SteD topology and location of mutants used in (D). Positions of blocks of alanine substitutions are shown as alternating blue and white stretches. Mutants impaired for binding to TMEM127 are located in transmembrane regions (red). Mutants defective for depletion of mMHCII surface levels are shown by ^∗^ (Bayer-Santos et al., 2016). (D) Coimmunoprecipitation of FLAG-TMEM127 with GFP-SteD mutants defective for depletion of mMHCII surface levels. HEK293ET cells expressing FLAG-TMEM127 and GFP-SteD alanine substitution mutants (as indicated in C) were lysed and proteins were immunoprecipitated with GFP-trap beads. Samples were analyzed by immunoblot using anti-GFP, anti-FLAG, and anti-tubulin (Tub) antibodies. Representative of 3 independent experiments. (E) Mander’s overlap coefficient of the fraction of TMEM127 positive pixels that colocalise with mMHCII positive pixels in the absence or presence of GFP-SteD. Data are representative of three independent experiments. Each dot represents the value for one cell. Error bars show mean ± SD.^∗^ p < 0.05 (Student’s t test). (F) Coimmunoprecipitation of FLAG-TMEM127 by anti-mMHCII (L243) antibody in absence or presence of GFP-SteD. Mel Juso cells stably expressing FLAG-TMEM127 or FLAG-TMEM127 and GFP-SteD were lysed and proteins were immunoprecipitated with L243 antibody. Samples were analysed by immunoblot using anti-DRα (MHCII), anti-GFP, anti-FLAG, anti-transferrin receptor (TfR), and anti-tubulin (Tub) antibodies. (G) Quantification of intensity of FLAG-TMEM127 signal in immunoprecipitates (F) relative to immunoprecipitated MHCII (DRα), in the absence or presence of GFP-SteD. Data show means ± SD from 5 independent experiments. ^∗^p < 0.05 (Student’s t test). See also Figures 4 and S3.

The requirement for TMEM127 and WWP2 for SteD-dependent di-ubiquitination of mMHCII suggested that SteD mediates an interaction between a TMEM127/WWP2 complex and mMHCII, resulting in its ubiquitination. After translocation from bacteria or following its ectopic expression, SteD localizes at the *trans*-Golgi network and in cytoplasmic vesicles that contain mMHCII (Bayer-Santos et al., 2016). Co-expression of GFP-SteD and FLAG-TMEM127 in Mel Juso cells showed that both proteins colocalize with mMHCII in vesicles (Figure 5A). In HEK293ET cells, GFP-SteD but not another tagged *Salmonella* effector (GFP-SifB), coprecipitated FLAG-TMEM127, showing that they can interact without MHCII (Figure 5B). To define region(s) of SteD required for binding to TMEM127 we used several alanine substitution mutants that are defective for depletion of mMHCII surface levels (Bayer-Santos et al., 2016). GFP-SteD mutants in the first and second transmembrane segments (SteD^Ala11, Ala12, Ala15, Ala16^) were defective for binding to FLAG-TMEM127 (Figures 5C and 5D), despite integrating normally into host cell membranes (Figure S5A). These results imply that SteD interacts with TMEM127 through its transmembrane domains. The reduced ability of these mutants to decrease mMHCII surface levels and of SteD^Ala16^ to cause di-ubiquitination of mMHCII (Bayer-Santos et al., 2016) is therefore correlated with their impaired binding to TMEM127.

Although SteD interacted with TMEM127 and TMEM127 was required for both SteD-mediated ubiquitination and surface depletion of mMHCII, microscopic analysis and co-immunoprecipitation of mMHCII and GFP-SteD from *TMEM127*^*−/−*^ Mel Juso cells revealed that their vesicular colocalization and physical interaction do not require TMEM127 (Figures S5B–S5E). Therefore, we questioned whether mMHCII was able to interact with TMEM127 and whether SteD was necessary for this interaction. TMEM127 colocalized extensively with mMHCII in vesicles, and this was not increased by co-expression of GFP-SteD (Figures 5A and 5E). However, while immunoprecipitation of mMHCII showed that it interacted only weakly with TMEM127, this was strikingly increased in the presence of GFP-SteD (Figures 5F and 5G). Therefore, while TMEM127 and mMHCII co-exist in the same vesicles and might interact to a small extent, their interaction is greatly stimulated or stabilized by SteD. Collectively these results suggest that SteD is an adaptor that can bind to both TMEM127 and mMHCII, thereby bridging these molecules and enabling ubiquitination of mMHCII.

### TMEM127- and WWP2-Dependent Ubiquitination of SteD

**Figure 6:**
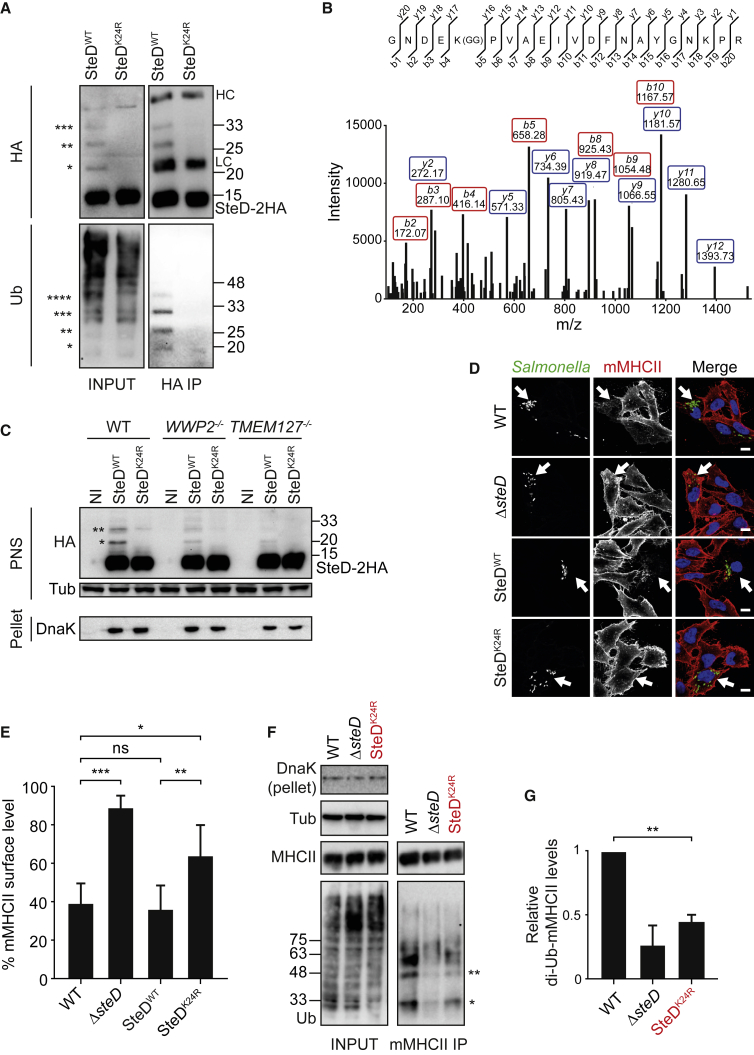
TMEM127- and WWP2-Dependent Ubiquitination of SteD K24 SteD variants expressed from a plasmid in *steD* mutant strains are indicated in black. SteD^K24R^ expressed from the chromosome is indicated in red. (A) SteD is ubiquitinated following translocation by intracellular *Salmonella*. Mel Juso cells were infected with *ΔsteD Salmonella* expressing HA-tagged WT SteD or K24 point mutant (SteD^K24R^) from a plasmid. Cells were lysed 20 h p.i. and proteins from the post-nuclear supernatant were immunoprecipitated with an HA antibody. Samples were analysed by immunoblot using anti-HA or anti-ubiquitin (Ub) antibodies. Bands with masses corresponding to mono, di-, tri-, and tetra-ubiquitinated SteD-2HA are indicated by ^∗^, ^∗∗^, ^∗∗∗^, and ^∗∗∗∗^, respectively. Protein size markers (kDa) are indicated on right. HC – IgG heavy chain. LC – IgG light chain. Representative of 3 independent experiments. (B) MALDI MS/MS spectrum of a peptide obtained after trypsinization of GFP-SteD obtained from Mel Juso cells stably expressing GFP-SteD using GFP-trap beads. K24 is modified with a G-G branch (K[GG]) indicating ubiquitination at this position. (C) WWP2- and TMEM127-dependent ubiquitination of SteD. WT or mutant Mel Juso cells were infected with *Salmonella* expressing HA-tagged WT SteD or SteD^K24R^ from a plasmid. At 20 h p.i., proteins were separated into pellet or post-nuclear supernatant (PNS) fractions and analysed by immunoblot using anti-HA, anti-tubulin (Tub), and anti-DnaK (as a marker for *Salmonella*) antibodies. Bands with masses corresponding to mono and di-ubiquitinated SteD-2HA are indicated by ^∗^ and ^∗∗^, respectively. Protein size markers (kDa) are indicated on right. Representative of 3 independent experiments. (D) SteD^K24R^ fails to deplete surface mMHCII. Representative confocal immunofluorescence microscopy images of Mel Juso cells infected with the indicated *Salmonella* strains expressing GFP (green). Cells were fixed 20 h p.i. and surface mMHCII was labelled with L243 antibody (red). Nuclei were stained with DAPI (blue). Arrows indicate infected cells. Scale bar, 10 μm. (E) Quantification of mMHCII surface levels in Mel Juso cells infected with the indicated *Salmonella* strains. Cells were analyzed by flow cytometry 20 h p.i., and amounts of surface mMHCII are expressed as a percentage of uninfected cells from the same sample. Data are from 3 independent experiments and show means ± SD. ^∗∗∗^ p < 0.001, ^∗∗^ p < 0.01, ^∗^ p < 0.05, ns, non-significant (one-way ANOVA followed by Tukey’s multiple comparison test). (F) An SteD^K24R^ chromosomal point mutant of *Salmonella* is defective for ubiquitination of mMHCII. Mel Juso cells infected with the indicated *Salmonella* strains were lysed 20 h p.i. and proteins were immunoprecipitated with L243 antibody. Samples were analyzed by immunoblot using anti-tubulin (Tub), and anti-DnaK (as a marker for *Salmonella*), anti-DRα (MHCII), and anti-ubiquitin (Ub) antibodies. Bands with masses corresponding to mono and di-ubiquitinated mMHCII β chain are indicated by ^∗^ and ^∗∗^, respectively. (G) Quantification of intensity of di-ubiquitinated mMHCII signal relative to wild-type-infected cells, from 3 experiments represented in (F). Data are means ± SD from 3 independent experiments. ^∗∗^ p < 0.01 (one sample t test). See also Figure S5.

Immunoblotting of SteD-2HA that had been translocated from bacteria into mammalian cells revealed a small proportion of the protein migrating with greater mass. This corresponded to mono-, di- and tri-ubiquitinated SteD (Figure 6A). Mass spectrometry analysis of GFP-SteD immunoprecipitated from the lysate of Mel Juso cells expressing GFP-SteD revealed the presence of a di-glycyl remnant on SteD K24 that is characteristic of ubiquitinated lysine residues (Figure 6B). No evidence of ubiquitination was found for the only other lysine in SteD, K38. Substitution of SteD K24 with arginine and expressed from bacteria in infected Mel Juso cells, abolished ubiquitination of SteD (Figure 6A). Ubiquitination of wild-type SteD was also much reduced in *TMEM127*^*−/−*^ and *WWP2*^*−/−*^ cells (Figure 6C). These results indicate that WWP2 ubiquitinates SteD (SteD-Ub) on K24 and that TMEM127 is essential for this.

### SteD Ubiquitination Enhances Its Activity

Next we investigated the functional significance of SteD-Ub. GFP-SteD^Ala5^ (incorporating K24A; Figure 5C) inserted normally into host membranes (Figures S5A and S6A) and interacted with TMEM127 (Figure 5D). However, Mel Juso cells infected with the *Salmonella* Δ*steD* mutant expressing and translocating HA-tagged SteD^K24R^ retained high surface levels of mMHCII, compared to cells infected with *Salmonella* translocating HA-tagged wild-type SteD (Figures 6D and 6E). We also constructed a chromosomal mutation encoding SteD^K24R^ and observed the same phenotype (Figure S6B). This was accompanied by significantly reduced ubiquitination of mMHCII in cells infected with *Salmonella* expressing SteD^K24R^ (Figures 6F and 6G). The ubiquitination of SteD is therefore important for its function in reducing mMHCII surface levels.

### SteD and mMHCII Undergo K63-Linked Ubiquitination

**Figure 7:**
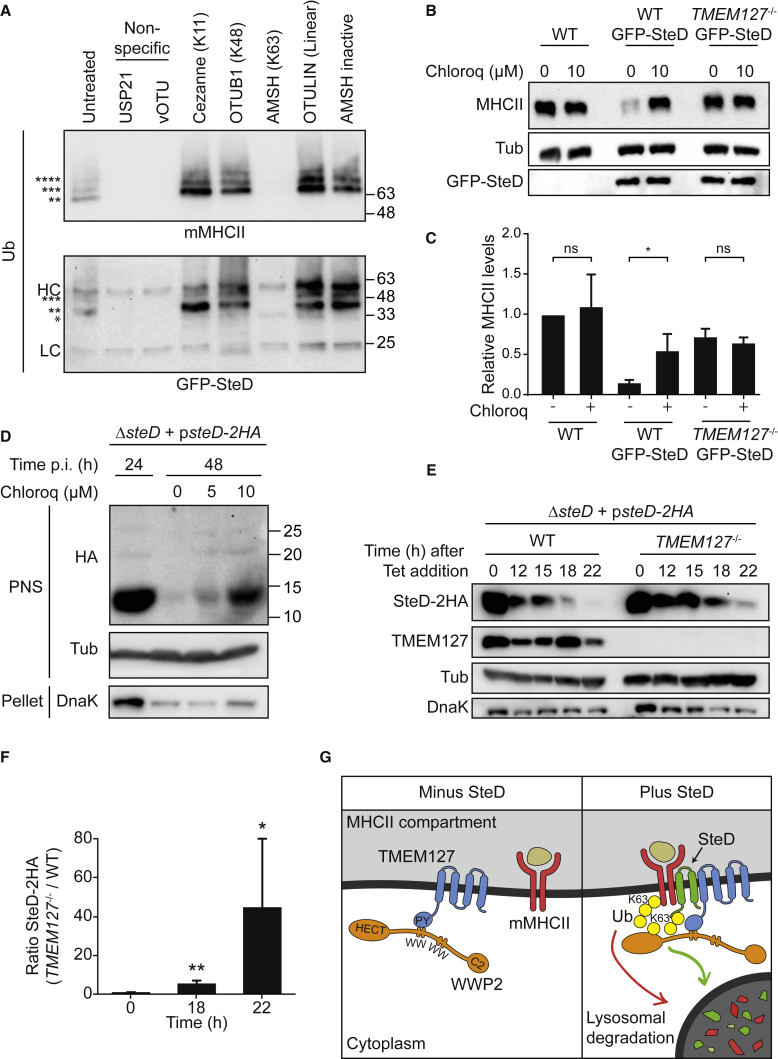
mMHCII and SteD Contain K63 Ubiquitin Linkages and Are Subject to Lysosomal Degradation (A) UbiCRest assay of ubiquitinated mMHCII and GFP-SteD collected from Mel Juso cells stably expressing GFP-SteD. Proteins were immunoprecipitated using L243 antibody (mMHCII) or GFP-trap, incubated with the indicated DUBs and analyzed by immunoblot using a pan-ubiquitin antibody. HC – IgG heavy chain. LC – IgG light chain. Number of ^∗^ corresponds to predicted number of ubiquitin molecules on SteD or mMHCII. Representative of 3 independent experiments. (B) Mel Juso cells (wild-type or *TMEM127*^*−/−*^), either non-transfected or expressing GFP-SteD, were mock-treated or exposed to chloroquine for 24 h. Cell lysates were analysed by immunoblot using anti-DRα (MHCII), anti-tubulin (Tub), and anti-GFP antibodies. (C) Quantification of intensity of DRα signal from 3 experiments represented in (B), normalized to that from WT Mel Juso cells in absence of chloroquine. Data are means ± SD. ^∗^ p < 0.05. ns, not significant (Student’s t test). (D) Mel JuSo cells were infected with *ΔsteD Salmonella* expressing SteD-2HA from a plasmid. Cells were lysed 24 h p.i. (left lane) or treated with indicated concentrations of chloroquine for an additional 24 h before lysis. Cell lysates were separated into pellet or post-nuclear supernatant (PNS) fractions and analyzed by immunoblot using anti-HA, anti-tubulin (Tub), or anti-DnaK (as a marker for *Salmonella*) antibodies. Representative of 3 independent experiments. (E) WT or *TMEM127*^*−/−*^ Mel Juso cells were infected with *ΔsteD Salmonella* expressing SteD-2HA from a plasmid. Bacteria were then killed with tetracycline (Tet) 20 h p.i., and the amount of SteD was analyzed at indicated time-points thereafter by immunoblot using anti-HA, anti-TMEM127, or anti-DnaK (as a marker for *Salmonella*) antibodies. (F) Quantification of intensity of SteD-2HA signal at indicated times post-Tet addition. Data are means ± SD. ^∗∗^ p < 0.01, ^∗^ p < 0.05 (one sample t test). (G) Model for mechanism of mMHCII surface depletion by SteD, TMEM127, and WWP2. TMEM127 (blue) interacts with the E3 ubiquitin ligase WWP2 (orange) via PPxY motif (PY) of TMEM127 and WW domain of WWP2. SteD (green) enables TMEM127 to interact with mMHCII (red), resulting in WWP2-dependent ubiquitination of mMHCII and SteD and their subsequent lysosomal degradation. See also Figure S6.

We used two complementary approaches to determine the type of ubiquitin linkages associated with mMHCII and SteD. In the UbiCRest assay (Hospenthal et al., 2015), ubiquitinated substrates are incubated with a panel of linkage-specific deubiquitinases (DUBs) to infer the type of chains attached to substrates. We used Mel Juso cells stably expressing GFP-SteD and collected mMHCII or GFP-SteD by immunoprecipitation (Figure 7A). These proteins were incubated with six DUBs with different specificities and were subjected to SDS-PAGE and immunoblotting using a pan-ubiquitin antibody. As expected, the non-specific DUBs USP21 and vOTU resulted in the cleavage of ubiquitin chains from both GFP-SteD and mMHCII. Of the DUBs that were active on K11-, K48-, K63-, or Met1-linked (linear) ubiquitin molecules (Figure S7A) only associated molecule with the SH3 domain of STAM (AMSH)-cleaved ubiquitin chains associated with GFP-SteD and mMHCII (Figure 7A), indicating that these proteins are modified primarily with K63-linked ubiquitin chains. In the second approach, the same samples were separated by SDS-PAGE, trypsinised and then subjected to ubiquitin-AQUA (absolute quantification) mass spectrometry (Kirkpatrick et al., 2006). This confirmed that the predominant ubiquitin chain type on both mMHCII and GFP-SteD is K63-linked (Figures S7B–S7E), consistent with previous work showing that WWP2 mainly catalyzes K63-linked ubiquitination (Jiang et al., 2015, Liao and Jin, 2010).

### Lysosomal Degradation of mMHCII and SteD

K63-linked ubiquitination of membrane proteins can lead to lysosomal degradation of substrates (Dores and Trejo, 2019, Duncan et al., 2006, Geetha et al., 2005). To assess lysosomal degradation of mMHCII, Mel Juso cells either expressing GFP-SteD or not, were exposed to the lysosomal inhibitor chloroquine and protein levels were examined 24 h later by immunoblotting. In untreated cells, GFP-SteD caused a strong reduction in total MHCII (Figures 7B and 7C). This was rescued either by chloroquine or lack of TMEM127 (Figures 7B and 7C). The amount of GFP-SteD was not noticeably affected by chloroquine or absence of TMEM127 (Figure 7B), possibly because of its overexpression after transfection. Therefore, we analysed the fate of SteD-2HA following its translocation from *Salmonella* in Mel Juso cells. At 24 h post-invasion bacteria were killed with tetracycline and cells containing dead bacteria were then mock-treated or exposed to chloroquine for a further 24 h. In mock-treated cells, SteD-2HA was virtually undetectable, but chloroquine (Figure 7D) or the absence of TMEM127 (Figures 7E and 7F) rescued a large fraction from degradation. Since TMEM127 is required for ubiquitination of SteD, SteD-Ub-induced ubiquitination of mMHCII and reduction of surface mMHCII (Figures 6E and 6F), we conclude that ubiquitination of SteD not only results (directly or indirectly) in its own lysosomal degradation, but importantly, enhances its activity.

## Discussion

MHCII surface levels in DCs are normally suppressed by the RING E3 ligase March-1 (Cho et al., 2015). Based on RNA knockdown and co-immunoprecipitation experiments, we previously proposed that in Mel Juso cells, SteD co-opts its homologue MARCH8 to ubiquitinate MHCII, thereby leading to its degradation (Bayer-Santos et al., 2016). However, in the current study, use of *MARCH* knockout cells failed to provide support for this, suggesting that the results in our previous study may have been caused by off-target effects of RNA interference. Therefore, it is not clear whether the direct or indirect interaction between SteD and MARCH8 is physiologically significant (Bayer-Santos et al., 2016). These findings led us to conduct genome-wide mutant screens for human proteins that are required for SteD function. Two screens revealed that TMEM127 and WWP2 are required by SteD to deplete surface mMHCII. One microRNA (mir-3150) was also strongly enriched in the two screens. However, three other gRNAs in the library targeting the same miRNA were not recovered above background level, suggesting it might be an off-target effect.

TMEM127 is involved in regulation of the mTORC1 lysosomal machinery (Deng et al., 2018) and insulin sensitivity (Srikantan et al., 2019) but its biochemical function was unknown. Mutations in *TMEM127* confer susceptibility to pheochromocytomas and renal cell carcinomas (Qin et al., 2010, Qin et al., 2014). WWP2 is a member of the NEDD4 family of HECT E3 ligases, of which there are 9 members in humans. Among those, WWP1 and ITCH form a very closely related subgroup with WWP2 (Jiang et al., 2015). Therefore, it is possible that partial redundancy might exist between these ligases (Martin-Serrano et al., 2005), providing an explanation for the finding that SteD activity was not completely abolished in cells lacking WWP2. However, although ITCH and WWP1 were each targeted by 6 guide RNAs in the two screens, virtually none were present in the sorted cell populations (Table S1). Therefore, it seems unlikely that loss of either ligase affects SteD function in otherwise wild-type cells.

NDFIP1 and NDFIP2 are integral membrane adaptors that interact, through their PPxY motifs, with NEDD4 E3 ligases including WWP2, thereby promoting degradation of transmembrane protein substrates (Foot et al., 2017). The presence of a PPAY sequence at the C terminus of TMEM127 and its requirement for binding WWP2 defines TMEM127 as a novel type of NDFIP. Our results in Figure 4 show that TMEM127 interacts with WWP2 in the absence of infection, but the identity of the endogenous substrate(s) of TMEM127 are currently unknown. One candidate is a component of the late endosomal/lysosomal adaptor and MAPK and MTOR activator (LAMTOR) complex, since the absence of TMEM127 is associated with increased levels of LAMTOR subunits and related signalling molecules (Deng et al., 2018). Our results raise the possibility that the higher levels of LAMTOR subunits in cells lacking TMEM127 are due to a reduction in their ubiquitination and lysosomal degradation. It is noteworthy that analysis of the mode of action of a *Salmonella* effector has provided a functional link between two previously unconnected mammalian proteins—a tumour suppressor and an oncogenic E3 ubiquitin ligase. The possibility that the broader effects resulting from mutation of the former (such as pheochromocytomas in humans [Qin et al., 2010] and defects in insulin signalling in mice and humans [Srikantan et al., 2019]) might be attributable to dysregulation of the latter, clearly deserves further investigation. Indeed, dysregulation of WWP2 has been associated with several types of cancer (Zhang et al., 2019) and WWP2 interacts with another tumour suppressor—phosphatase and tensin homolog (PTEN) (Maddika et al., 2011). Our results did not provide any evidence for the involvement of TMEM127 or WWP2 in mMHCII regulation in DCs in the absence of SteD. However, since NEDD4 family members including WWP2 are involved in T cell function (Aki et al., 2018), it would be interesting to determine whether TMEM127 has endogenous substrates that affect antigen presentation and/or T cell activation.

Regardless of their endogenous functions, it is clear from this study that SteD appropriates TMEM127 and WWP2 to promote ubiquitination and degradation of both itself and mMHCII. Immunofluorescence microscopy confirmed that TMEM127 localizes within endosomal compartments (Qin et al., 2014) and established that a substantial proportion of these contain mMHCII. Expression of SteD did not cause a noticeable redistribution of TMEM127, and neither was TMEM127 required for co-localisation between SteD and mMHCII, suggesting that following its translocation from bacterial vacuoles, SteD is recruited to compartments that already contain both mMHCII and TMEM127. Our results imply that SteD and TMEM127 interact through their transmembrane regions and previous work indicates that the cytoplasmic C-terminal domain of SteD is important for its interaction with mMHCII (Bayer-Santos et al., 2016). Lack of TMEM127 did not affect interactions between SteD and mMHCII, and lack of MHCII did not affect interactions between SteD and TMEM127. However, SteD enhanced the interaction between TMEM127 and mMHCII. Therefore, we conclude that SteD is an adaptor that bridges these molecules (Figure 7G).

Analysis of SteD-induced ubiquitin linkages showed that both mMHCII and SteD are modified predominantly by K63-linkages, consistent with previous work on WWP2 (Jiang et al., 2019, Mund et al., 2015, Xu et al., 2004). K11 linkages were also detected by ubiquitin-AQUA mass spectrometry, suggesting the presence of mixed K63/K11 chains. Interestingly, such mixed chains are generated on MHCI molecules by an E3 ligase of Kaposi’s sarcoma-associated herpesvirus, leading to endocytosis of MHCI (Boname et al., 2010). Our finding that degradation of both proteins was prevented by chloroquine or absence of TMEM127 is also consistent with other work showing that K63-linked ubiquitin is a signal for recognition by ESCRT complex components, internalization into multivesicular bodies (MVBs) (Huang et al., 2013, Lauwers et al., 2009) and lysosomal degradation. We showed previously that SteD induces ubiquitination on K225 of the β chain of MHCII (Bayer-Santos et al., 2016). Although the two lysines of SteD—K24 and K38—are both part of the cytoplasmic N-terminal domain, TMEM127-dependent ubiquitination occurred exclusively on K24. This selectivity implies a highly specific structural organization of SteD in relation to mMHCII and the TMEM127/WWP2 complex. The K24R point mutant of SteD was impaired in ubiquitination and surface depletion of mMHCII, indicating that ubiquitination of SteD has an important functional role. One possibility is that the amplified mass of SteD-Ub (approximately 28 kDa for the di-ubiquitin species, compared to 11 kDa for SteD) provides a scaffold that facilitates the arrangement of a higher order complex containing mMHCII, SteD, TMEM127, WWP2 together with its cognate E2 conjugating enzyme, and which favours ubiquitination of mMHCII. Alternatively, SteD-Ub might increase the localised concentration of K63-linked ubiquitin, amplifying a signal that leads to degradation of both mMHCII and itself. In addition to linking TMEM127/WWP2 with a novel substrate (mMHCII), SteD might also contribute to the activation of autoinhibited WWP2 (Chen et al., 2017, Mund et al., 2015). Addressing these questions will probably require a cell-free assay for SteD activity and deeper mechanistic insights will necessitate structural analysis of SteD together with relevant regions of other proteins.

It is remarkable that degradation of mMHCII can be controlled endogenously by a MARCH RING E3 ligase and exogenously by a NEDD4 HECT E3 ligase, and that the latter process involves ubiquitination of the inducing bacterial effector, augmenting both its activity and (either directly or indirectly) its degradation. Very little is known about processes through which bacterial pathogens interfere directly with antigen presentation. The fact that *Salmonella* evolved an effective mechanism that depletes peptide-loaded surface MHC class II molecules shows that CD4^+^ T cell-mediated adaptive immunity has exerted a strong selective pressure on this pathogen and suggests that other sophisticated mechanisms remain to be discovered in other bacteria whose virulence is controlled by antigen presentation and T cell responses.

## STAR★Methods

### Key Resources Table

**Table:** 

REAGENT or RESOURCE	SOURCE	IDENTIFIER
**Antibodies**		

Mouse monoclonal anti-HLA-DR (cloneL243)	Sigma-Aldrich	Cat#SAB4700731
Mouse monoclonal anti-HLA-DR alpha chain (clone TAL.1B5)	DAKO	Cat#M0746; RRID: AB_2262753
Mouse monoclonal anti-DnaK (clone 8E2/2)	ENZO	Cat#ADI-SPA-880-F; RRID: AB_10619012
Goat polyclonal anti-Common *Salmonella* Antigens	KPL	Cat#01-91-99
Rabbit Monoclonal anti-GFP	Invitrogen	Cat#G10362
Mouse monoclonal anti anti-ubiquitin (clone P4D1)	Santa Cruz	Cat#sc-8017; RRID: AB_628423
Mouse monoclonal anti-tubulin	DSHB	Cat#E7
Rabbit polyclonal anti-actin	Sigma-Aldrich	Cat#A2066; RRID:AB_476693
Mouse monoclonal anti-CRISPR-Cas9	Novus Biological	Cat#NBP2-36440
Mouse monoclonal anti-Golgin97 (clone CDF4)	eBioscience	Cat#14-9767-82
Mouse monoclonal anti-Transferrin receptor (H68.4)	Zymed (S. Meresse)	Cat#13-6800
Mouse monoclonal anti-FLAG (clone M2)	Merck	Cat#F3165-2MG
Rabbit polyclonal anti-FLAG	Merck	Cat#F7425
Mouse monoclonal anti-HA11 (clone 16B12)	Biolegend	Cat#901502; RRID: AB_2565007
Rabbit polyclonal anti-TMEM127	Bethyl laboratories	Cat#A303-450A; RRID: AB_10952702
Rabbit polyclonal anti-WWP2	Abcam	Cat#ab103527; RRID: AB_10710285
Mouse monoclonal anti-MHCII-APC (clone M5/114.15.2)	Miltenyi Biotec	Cat#130-102-898; RRID: AB_2660057
Hamster monoclonal anti-CD11c-VioBlue (clone N418)	Miltenyi Biotec	Cat#130-102-797; RRID: AB_2660157
Rat monoclonal anti-CD4-PerCP-Cy5.5 (clone RM4-5)	BD Biosciences	Cat#550954; RRID: AB_393977
Hamster monoclonal anti-CD3ε-PE-Vio770 (145-2C11)	Miltenyi Biotec	Cat#130-102-794; RRID: AB_2660399

**Bacterial and Virus Strains**		

*Salmonella enterica* serovar Typhimurium 12023	NCTC	NCTC 12023
*Salmonella enterica* serovar Typhimurium 12023 ΔsteD::km	Bayer-Santos et al., 2016	N/A
*Salmonella enterica* serovar Typhimurium 12023 ΔsteD::km + pSteD-2HA-SrcA	Godlee et al., 2019	N/A
*Salmonella enterica* serovar Typhimurium 12023 + pFCcGi	This study	N/A
*Salmonella enterica* serovar Typhimurium 12023 ΔsteD::km + pFCcGi	This study	N/A
*Salmonella enterica* serovar Typhimurium 12023 ΔsteD::km + pSteD-2HA-SrcA + pFCcGi	This study	N/A
*Salmonella enterica* serovar Typhimurium 12023 ΔsteD::km + pSteD^K24R^-2HA-SrcA	This study	N/A
*Salmonella enterica* serovar Typhimurium 12023 ΔsteD::SteD^K24R^	This study	N/A
*E. coli* DH5α	Thermo Fisher Scientific	Cat#18265017

**Chemicals, Peptides, and Recombinant Proteins**		

Chloroquine diphosphate salt	Sigma-Aldrich	Cat#C6628-25G; CAS: 50-63-5
Enzymes for UbiCRest	Hospenthal et al., 2015	N/A
Blasticidin S	Merck	Cat#15205-25MG; CAS: 3513-03-9
Puromycin dihydrochloride	Sigma-Aldrich	Cat#P8833-25MG; CAS: 58-58-2
Carbenicillin disodium salt	Sigma-Aldrich	Cat#C1389-5g; CAS: 4800-94-6
Chloramphenicol	Merck	Cat#C0378-25G; CAS: 56-75-7
Kanamycin sulfate from Streptomyces kanamyceticus	Sigma-Aldrich	Cat#K1377-5g; CAS: 25389-94-0
5(6)-Carboxyfluorescein diacetate N-succinimidyl ester	Sigam-Aldrich	Cat#21888-25MG-F; CAS: 150347-59-4
Ovalbumin (323-339) (chicken, Japanese quail)	Sigam-Aldrich	Cat#O1641-1MG

**Experimental Models: Cell Lines**		

Hek293T	Provided by F. Randow	N/A; RRID: CVCL_0063
Hybridoma L243	Provided by S. Meresse	N/A; RRID: CVCL_4533
MelJuSo	Provided by J. Neefjes	N/A; RRID: CVCL_1403
MelJuSo CRISPR/Cas9 MARCH8 KO	This study	N/A
MelJuSo CRISPR/Cas9 TMEM127 KO	This study	N/A
MelJuSo CRISPR/Cas9 WWP2 KO	This study	N/A
MutuDCs	Provided by A. Orbea	Fuertes Marraco et al., 2012
MutuDCs CRISPR/Cas9 MARCH1 KO	Provided by J. Mintern	Wilson et al., 2018
MutuDCs CRISR/Cas9 TMEM127 KO	This study	N/A
MutuDCs CRISR/Cas9 WWP2 KO	This study	N/A

**Experimental Models: Organisms/Strains**		

OT-II mice	Charles River	C57BL/6-Tg(TcraTcrb)425Cbn/Crl; RRID: IMSR_CRL:643

**Oligonucleotides**		

See Table S2 for primer sequences	N/A	N/A

Recombinant DNA		

Plasmid: GFP-SteD	Bayer-Santos et al, 2016	N/A
Plasmid: pMD-GAGPOL	Provided by F. Randow	Randow and Sale, 2006
Plasmid: VSVG	Provided by F. Randow	Randow and Sale, 2006
Plasmid: lentiCas9-Blast	Sanjana et al, 2014 Gift from Feng Zeng	Addgene Plasmid #52962
Plasmid: psPAX2	Gift from Didier Trono	Addgene Plasmid #12260
Plasmid: pMD2.G	Gift from Didier Trono	Addgene Plasmid #12259
Plasmid: lentiguide-puro	Sanjana et al, 2014 Gift from Feng Zhang	Addgene Plasmid #52963
Pooled plasmid library: Human CRISPR knockout Pooled Library (GeCKO v2)	Sanjana et al., 2014 Gift from Feng Zhang	Addgene Plasmid #1000000048
WWP2 gene sequence	Genscript	OHu14116
TMEM127 gene sequence	genscript	OHu10702

**Software and Algorithms**		

Biopython	Biopython	https://biopython.org
Count_spacers.py	Sanjana et al, 2014	https://github.com/fengzhanglab/Screening_Protocols_manuscript/blob/master/count_spacers.py
CRISPRAnalyzer	CRISPRAnalyzeR	http://crispr-analyzer.dkfz.de/
CRISPR Design Tool	Dharmacon	https://horizondiscovery.com/en/products/tools/CRISPR-Design-Tool
Prism v8	GraphPad	https://www.graphpad.com/scientific-software/prism/
FIJI	ImageJ	https://fiji.sc/
Coloc 2 ImageJ plugin	ImageJ	http://imagej.net/Coloc_2
Image Lab	BioRad	https://www.bio-rad.com/en-uk/product/image-lab-software?ID=KRE6P5E8Z
FlowJo v10	BD Life Sciences	https://www.flowjo.com/

**Other**		

Laser Scanning Microscope 710	Zeiss	N/A
BD LSRFortessa™	BD Biosciences	N/A
BD FACSAria™ III	BD Biosciences	N/A
Dionex Ultimate 3000 HPLC system	Thermo Fisher Scientific	N/A
Q Exactive™	Thermo Fisher Scientific	N/A

### Resource Availability

#### Lead Contact

Further information and requests for resources and reagents should be directed to and will be fulfilled by the Lead Contact, David W. Holden (d.holden@imperial.ac.uk).

#### Materials Availability

Cell lines, plasmids and *Salmonella* strains generated in this study are freely available by requests directed to the lead contact.

#### Data and Code Availability

The published article includes all datasets generated or analyzed during this study.

### Experimental Model and Subject Details

#### Bacterial Strains

Bacteria were grown in Luria–Bertani (LB) medium supplemented with carbenicillin (50 μg mL^-1^), kanamycin (50 μg mL^-1^) or chloramphenicol (30 μg mL^-1^) as appropriate. See Key Resources Table for all bacterial strains.

#### Cell Culture

HEK293ET cells and human Mel Juso cells were maintained in Dulbecco’s modified eagle medium (DMEM; Sigma) supplemented with 10% heat-inactivated fetal calf serum (FCS; Gibco, Life Technologies) at 37°C in 5% CO_2_. MutuDCs (Fuertes Marraco et al., 2012) and March1^-/-^ MutuDCs were maintained in IMDM-glutamax (GIBCO 31980), supplemented with 8–10% heat inactivated, endotoxin-free FCS, 10 mM HEPES pH 7.4, 50 μM β-mercaptoethanol and 50 U mL^-1^ of penicillin and 50 μg mL^-1^ streptomycin. T cells expressing OVA-specific T cell receptor were isolated from cell suspensions of spleens and lymph nodes of female OT-II (C57BL/6-Tg(TcraTcrb)425Cbn/Crl) mice (Charles River) by magnetic sorting of CD4^+^ cells (Miltenyi Biotec) and labeled with Carboxyfluorescein succinimidyl ester (CFSE) as described previously (Quah and Parish, 2010).

#### Cell Infection

For infection of Mel Juso cells, overnight Luria broth (LB) cultures of *S.* Typhimurium strains were diluted 1:33 in LB and incubated with shaking at 37°C for 4 h before being added to cells at an MOI of 100:1 for 30 min. Cells were washed 3 times with PBS and incubated in fresh medium containing gentamicin (100 μg mL^-1^) for 1 h to kill extracellular bacteria. After 1 h, the antibiotic concentration was reduced to 20 μg mL^-1^, and cells were processed 20 h post-invasion (p.i.). For infection of MutuDCs, *S.* Typhimurium strains from overnight cultures were added to cells at an MOI of 20:1, cells were centrifuged at 110 *g* for 5 min and incubated at 37°C for 30 min. Cells were washed 3 times with PBS and incubated in fresh medium containing gentamicin (100 μg mL^-1^) for 1 h to kill extracellular bacteria. After 1 h, the antibiotic concentration was reduced to 20 μg mL^-1^, and the cells were processed 20 h post-invasion (p.i.).

#### Mouse Strain

Female C57BL/6 OT-II mice (Charles River) were housed as 5 mice per ventilated cage under Specified Pathogen Free conditions. T cells were extracted from spleens and lymph nodes of 8-12 week old animals.

#### Mouse Ethics Statement

Experiments involving cells from OT-II mice were conducted in accordance with European Directive 2010/ 63/EU regulations with approval from Imperial College London, Animal Welfare and Ethical Review Body (ICL AWERB) under the Personal Project license of David Holden.

### Method Details

#### Plasmid Construction

See Table S2 for all primer sequences. DNA sequences encoding WWP2 and TMEM127 genes were obtained from Genscript (#OHu14116D and #OHu10702D respectively). GFP-WWP2-, GFP-TMEM127- and FLAG-TMEM127-expressing plasmids were obtained as follows: *TMEM127* was amplified by PCR using *TMEM127*-BsmBI_NcoI_Flag-F and *TMEM127*-BsmBI_NotI-R primers or *TMEM127*-BsmBI_PciI_F and *TMEM127*-BsmBI_NotI-R primers. PCR fragments were then digested by BsmBI and ligated into M6P or GFP-M4P lentiviral plasmids, respectively, following their digestion with NcoI/NotI or PciI/NotI. *WWP2* was amplified by PCR with primers WWP2-BsmBI_PciI_F and WWP2-BsmBI_NotI-R. PCR fragments were then digested by BsmBI and ligated into M5P lentiviral plasmid (Randow and Sale, 2006) following its digestion with NcoI/NotI. Flag-TMEM127 Y236A was obtained using *TMEM127*_PY_BsmBI_NotI-R primer containing the mutation. The *steD* mutation encoding K24R was introduced in plasmid pSteD-2HA-SrcA (Godlee et al., 2019) by overlapping PCR (Heckman and Pease, 2007). Two PCR fragments were obtained using primers ovK24R-F/SteD-Sac-R and ovK24R-R/SteD-Hind-F respectively. A second PCR was then done with the two amplicons as templates using primers SteD-Sac-R and SteD-Hind-F. The resulting DNA fragment and pSteD-2HA-SrcA were digested with HindIII and SacI before ligation and transformation in DH5α.

Lentiviral plasmids expressing gRNAs for directed CRISPR/Ca9 mutagenesis were obtained by ligation of duplexed DNA primers (sequences are shown in Table S2). For the generation of *MARCH8*^*-/-*^, *TMEM127*^*-/-*^ and *WWP2*^*-/-*^ Mel JuSo cells, duplexed primers were introduced into lentiguide-puro (Addgene #52963) by ligation following its digestion by Esp3I. For the generation of *Tmem127*^*-/-*^ and *Wwp2*^*-/-*^ MutuDCs, duplexed primers were introduced in lentiCRISPRv2 (Addgene #52961) by ligation following its digestion by Esp3I.

#### Transfection and Virus Production

HEK293ET cells were seeded 24 h before transfection. DNA transfection procedures were carried out using Lipofectamine 2000 according to the manufacturer’s protocol (Life Technologies). Plasmids and lipofectamine 2000 were incubated in OptiMEM for 5 min at room temperature. Both solutions were combined and incubated for 20 min at room temperature before being added to cells. For coimmunoprecipitation experiments, HEK293ET cells were seeded in 6-well plates and were transfected with 0.5 μg DNA and 2 μL lipofectamine per well. Cells were then incubated for 24 h at 37°C, 5% CO_2_ before lysis.

Lentiviruses for transduction were produced in HEK293ET cells by cotransfection. The lentiviral expression vector M6P encoding FLAG-TMEM127 or FLAG-TMEM127^Y236A^ and the lentiviral expression vector M4P encoding GFP-SteD, GFP-WWP2 or GFP-TMEM127 were cotransfected together with the packaging plasmids VSVG and GagPol. The lentiviral expression vector lentiguide-puro (Addgene #52963), lentiCas9-Blast (Addgene #52962) or lentiCRISPRv2 (Addgene #52961) were co-transfected together with the packaging plasmids psPAX2 and pMD2.G. In both cases, the culture medium was replaced 24 h after transfection and the supernatant containing viruses was collected 48 h after transfection and filtered.

#### Generation of Stable Cell Lines

To generate Mel JuSo cells stably expressing FLAG-TMEM127, FLAG-TMEM127^Y236A^, GFP-SteD, GFP-WWP2 or GFP-TMEM127, lentiviruses were added to Mel JuSo cells together with polybrene at 8 μg mL^-1^. At 24 h post transduction, cells were either selected with puromycin (0.8 μg mL^-1^), hygromycin (750 μg mL^-1^) or sorted by flow cytometry for the GFP constructs.

#### CRISPR/Cas9 Genome-Wide Mutant Screen

A clonal Mel JuSo cell line stably expressing GFP-SteD was first isolated and expanded. This cell line was transduced with lenti-Cas9-Blast lentivirus at an MOI of 0.3. Two days post-transduction, Blasticidin selection was carried out at 5 μg mL^-1^. A clonal population of GFP-SteD-, Cas9-expressing Mel JuSo cells was then selected and Cas9 expression was confirmed by immunoblot using anti-N-Terminal CRISPR-Cas9 antibody. The gRNA GeCKO v2.0 plasmid libraries (A and B pooled) (Addgene #1000000049, generated by the Feng Zhang laboratory) was packaged in lentivirus by co-transfection in HEK293ET cells with pMD2.G and psPAX2 (Sanjana et al., 2014). A total of approximately 10^8^ Cas9-, GFP-SteD- expressing Mel Juso cells were transduced with the lentivirus library at a MOI of 0.3 and 48 h later puromycin selection was applied at 0.8 μg mL^-1^. One week later, cells were detached and labelled with L243 antibody (1:300 dilution in sterile FACS buffer (5 % FCS and 1 mM EDTA in PBS) for 30 min) and secondary anti-mouse Alexa647 (1:300 dilution in sterile FACS buffer for 30 min). The top 1% GFP^high^/L243^high^ cells were sorted using a FACS Aria III high speed cell sorter. The sorting process was repeated twice after a week of expansion. Genomic DNA was extracted (Wizard, Promega) from combined sorted cells (approximately 2 x 10^7^ cells) and an equivalent number of unsorted mutagenized pooled cells grown for the same amount of time. Genomic DNA from sorted or unsorted cells were used as templates for PCRs to amplify the gRNAs using primers described by (Joung et al., 2017) (Table S2). PCR products were pooled and purified by agarose gel extraction and subjected to deep sequencing by Illumina MiSeq at the Imperial College BRC Genomics Facility. Quantification of reads was done using the count_spacers.py. program in Python (Joung et al., 2017) and statistical analyses were carried out using CRISPRAnalyzer (http://crispr-analyzer.dkfz.de/). When mMHCII is affected by SteD, Mel Juso cells grow a little more slowly, explaining the enrichment of *TMEM127* and *WWP2* mutant cells in the non-sorted (control) population (Table S1).

#### CRISPR/Cas9 Targeted Mutant Construction

gRNAs for human *MARCH8* (GGCAGGCCTGGTGCACGAAG) and mouse *Tmem127* (CGTGCTGGGCTATGTAAACC) and *Wwp2* (ACTGCTTTGGTGGCAGATCC) were designed using software available on the Dharmacon website (https://dharmacon.horizondiscovery.com/gene-editing/crispr-cas9/crispr-design-tool/). The two most abundant gRNA hits from the screen targeting human *TMEM127* (HGLibB_50855 and HGLibA_58204) and *WWP2* (HGLibA_62743 and HGLibA_62741) (Table S1) were selected to create *TMEM127* and *WWP2* knockouts in Mel JuSo cells. Screen results were validated using knockouts made with both gRNAs. Knockout clones made using HGLibB_50855 (TMEM127) and HGLibA_62743 (WWP2) were used for all further experiments (Figure S2). The gRNA sequences were ligated into a lentiviral plasmid as described above (plasmid construction).

To generate mutants of *MARCH8*, *TMEM127* and *WWP2* in Mel JuSo cells, cells were first transduced with lentiCas9-Blast viruses. After blasticidin selection (5 μg mL^-1^), single clones were isolated and Cas9 expression verified by immunoblot. Cas9-expressing Mel JuSo cells were then transduced with virus encapsulating lentiGuide-puro-gRNA. After puromycin selection (0.8 μg mL^-1^), single clones were isolated and gene inactivation was verified by genomic DNA sequencing and (with exception of *MARCH8*, for which a specific antibody was not available) immunoblot using rabbit polyclonal anti-TMEM127 or anti-WWP2 antibodies. To generate *Tmem127* and *Wwp2* MutuDC mutants, cells were transduced with virus encapsulating lentiCRISPRv2-gRNA (encoding both Cas9 and corresponding gRNAs). After puromycin selection, single clones were isolated and gene inactivation was verified by genomic DNA sequencing and immunoblot.

#### Immunofluorescence Microscopy

Cells were seeded onto coverslips and infected as described above. At 20 h p.i. cells were washed in PBS, fixed in 3 % paraformaldehyde in PBS for 15 min at room temperature, then the paraformaldehyde was quenched by incubation with 50 mM NH_4_Cl for 10 min. For surface labelling of mMHCII, primary and secondary antibodies were diluted in 10% horse serum (Sigma) and coverslips were washed in PBS. For intracellular labeling all antibodies were diluted in 10 % horse serum and 0.1 % saponin in PBS and coverslips were washed in 0.1 % saponin in PBS. Coverslips were incubated with appropriate primary antibodies for 1 h at room temperature, washed in PBS, then incubated with secondary antibodies for 1 h at room temperature. Finally, where used, coverslips were incubated with DAPI for 5 min, washed in PBS then mounted onto glass slides using Aqua-Poly/Mount (Polysciences). For selective permeabilisation, digitonin treatment was carried out on live cells. Coverslips were placed on ice, washed with KHM buffer (110 mM KOAc, 20 mM HEPES, 2 mM MgCl_2_, pH 7.3) and incubated for 5 min with 25 μg/mL digitonin diluted in KHM. Coverslips were then washed and incubated with primary antibodies diluted in 10% FCS in PBS for 30 min on ice. After washes, cells were fixed. For Triton X-100 permeabilisation cells were fixed and then incubated with 0.1% Triton X-100 for 5 min at room temperature prior to labelling for 30 min with primary antibodies diluted in 10% FCS in PBS. For both detergent treatments coverslips were incubated with secondary antibodies at room temperature under standard procedures. Coverslips were imaged using an LSM 710 inverted confocal laser-scanning microscope (Zeiss GmbH).

#### Image Analysis

Image analyses were done with ImageJ software. Pearson’s correlation coefficient was used to quantify the colocalisation of GFP-WWP2 to FLAG-TMEM127 (wild-type or mutant). The extracellular background was subtracted from images using the Background Subtraction function in ImageJ, with a rolling ball radius equal to 200 pixels or 26.4 μm. Pearson’s correlation coefficient values were obtained from individual cells using the Coloc 2 ImageJ plugin (http://imagej.net/Coloc_2).

Manders’ colocalization coefficient was used to measure the proportion of colocalising pixels between two punctate signals. The extracellular background was subtracted from images using the Background Subtraction function in ImageJ, with a rolling ball radius equal to 200 pixels or 26.4 μm. Local background was corrected by subtracting the median intensity of a 10 x 10 pixel region surrounding each pixel. Non-specific fluorescence was then subtracted using values measured from unlabelled cells. The images were then converted to binary and the Manders’ colocalization coefficient was measured from individual cells using the Coloc 2 imageJ plugin.

#### Flow Cytometry

Surface levels of mMHCII or MHCII on Mel Juso or MutuDC cells respectively, were measured following infection as described previously (Bayer-Santos et al., 2016), with minor modifications. In brief, Mel Juso cells were detached from cell culture plates using 2 mM EDTA in PBS. All antibodies were diluted in FACS buffer. Cells were labelled with mouse anti-mMHCII primary antibody (clone L243 for Mel Juso) or anti-MHCII (I-A/I-E, clone M5/114, APC-conjugated for MutuDC) at 1:300 dilution for 30 min on ice, washed in cold PBS, then labelled with donkey anti-mouse secondary antibody (no secondary antibody for MutuDC) at 1 :300 dilution for 30 min on ice. After washing with cold PBS, cells were fixed in 3.7% paraformaldehyde for 1 h at room temperature. Data were acquired using a Fortessa flow cytometer (BD Biosciences) and analysed using FlowJo v10 software. Surface levels of mMHCII and MHCII were calculated as median fluorescence of infected cells /median fluorescence of non-infected cells × 100.

Proliferation of OVA-specific CD4^+^ T cells was measured after incubation with anti-CD3ε (clone 145-2C11), anti-CD4 (clone RM4-5), anti-CD11c (clone N418) and anti-MHCII (I-A/I-E, clone M5/114) antibodies for 30 min on ice. All antibodies were diluted 1:200. Gates were set up to distinguish between MutuDC (CD11c^high^, CD3ε-) and CD4^+^ T cells (CD11c^low^, CD3ε^+^, CD4^+^). Infection rates and surface levels of MHCII were measured on MutuDCs as described above. Proliferation of CD4^+^ T cells was calculated as % of CFSE^low^ CD4^+^ T cells upon normalization to control sample containing no OVA peptide.

#### Membrane Fractionation

Membrane fractionation was carried out as previously described (Bayer-Santos et al., 2016). Mel Juso cells expressing GFP-tagged SteD variants were collected and lysed in homogenization buffer (250 mM sucrose, 3 mM imidazole (pH 7.4), and 1 mM PMSF) by mechanical disruption using a Dounce homogenizer. The post-nuclear supernatant was collected after centrifugation at 1,800 *g* for 15 min. The membrane fraction was pelleted by centrifugation at 100,000 g for 1 h at 4°C. To remove peripherally-associated proteins, the pellet was resuspended in 2.5 M urea and incubated for 15 min on ice followed by centrifugation at 100,000 g for 1 h at 4°C. This yielded a second pellet containing integral membrane proteins and a supernatant containing peripherally-associated membrane proteins. Pellets were resuspended in homogenisation buffer and all samples were analysed by SDS-PAGE and immunoblotting using anti-HLA-DR alpha chain, anti-GFP, anti-Golgin97 and anti-actin antibodies. The MHCII α chain was used as an integral membrane protein control. Actin was used as a soluble protein control. Golgin97 was used as a peripherally-associated membrane protein control.

#### Coimmunoprecipitations

HEK293ET or Mel Juso (wild-type or knockout) expressing GFP-SteD, GFP-WWP2, GFP-SifB, FLAG-TMEM127 or infected with *Salmonella* strains as indicated were resuspended in PBS containing 5 mM EDTA and washed once in PBS. Cells were lysed in lysis buffer (150 mM NaCl, 50 mM Tris pH 7.4, 5 mM EDTA, 0.5% Triton X100, 5% glycerol, 10 mM iodoacetamide and protease inhibitor cocktail tablets (Roche) for 10 min at 4°C. The post-nuclear supernatant was isolated by centrifugation at 16,000 *g* for 15 min. Proteins were immunoprecipitated by incubation with CNBr sepharose-coupled L243 antibody, anti-HA sepharose beads (Pierce) or anti-GFP-Trap beads (ChromoTek) for 2 h at 4°C. Immunoprecipitates were washed four times with lysis buffer and boiled in SDS buffer at 95°C for 5 min before analysis by SDS-PAGE and immunoblotting using anti-HLA-DR alpha chain, anti-GFP, anti-tubulin, anti-transferrin receptor, anti-HA, anti-TMEM127, anti-WWP2, and anti-FLAG antibodies. For ubiquitin immunoblots immunoprecipitates were eluted with 100 mM glycine (pH 3.0) and probed with mouse monoclonal anti-ubiquitin antibody. Immunoblots were visualised using ECL detection reagents (GE Healthcare, Thermo Scientific) on a Chemidoc™ Touch Imaging System (Bio-Rad) and densitometry measurements were carried out using Image Lab software (Bio-Rad).

#### Construction of SteD^K24R^ Chromosomal Mutation

See Table S2 for primer sequences. Chromosomal allelic exchange was used to construct the *steD*^K24R^ *Salmonella* mutant. The suicide plasmid pGP704-*steD*^K24R^ was constructed by overlap PCR (Heckman and Pease, 2007) using primers pGP704-steD-R and ovK24RF, pGP704-steD-F and ovK24RR then ligated into pGP704 (Miller and Mekalanos, 1988). To insert the I-SceI recognition site into the *steD* ORF, the pWRG100 plasmid (Blank et al., 2011) was used as a template to amplify I-SceI recognition site along with the chloramphenicol resistance cassette using primers pWRG100_dsteD_F and pWRG100_dsteD_R. The PCR product was transformed into pKD46-containing *S.* Typhimurium 12023 expressing λ Red recombinase by electroporation (Datsenko and Wanner, 2000) to make strain *S.* Typhimurium 12023 (SteD I-SceI). Plasmid pGP704-*steD*^K24R^ was transferred by conjugation from *E. coli* S17-1 λ pir to *S.* Typhimurium 12023 (SteD I-SceI). Exconjugants were selected by growth on chloramphenicol and carbenicillin. Successful recombinants were selected by expression of I-SceI endonuclease (Blank et al., 2011) and lack of growth on chloramphenicol and carbenicillin. Positive clones were verified by sequencing.

#### Preparation of UbiCRest Deubiquitinases

DUBs used in the UbiCRest analysis were expressed and purified as in (Hospenthal et al., 2015). Briefly, USP21 (196-565 in pOPIN-S), vOTU (1-183 in pOPIN-K), Cezanne (53-446 in pOPIN-K), OTUB1^∗^ (UBE2D2-OTUB1 fusion protein (Hospenthal et al., 2015, Michel et al., 2015) in pOPIN-B), AMSH^∗^ (STAM2-AMSH fusion protein (Michel et al., 2015) in pOPIN-B), inactive AMSH^∗^ (E280A mutation), and OTULIN (1-352 in pOPIN-B) were transformed into Rosetta2 (DE3) pLacI *E. coli*. The resulting transformants were used to inoculate 2 x TY media and grown at 37°C until an optical density at 600 nm of 0.6-0.8 was reached, at which point the cultures were cooled to 18°C and induced with 0.2 mM IPTG for 18 h of expression. Cells were harvested, resuspended, and lysed by sonication. Purification was carried out according to manufacturer’s suggestions using either HisPur Cobalt resin (Thermo Fisher) for pOPIN-B and pOPIN-S constructs or Glutathione Sepharose 4B (GE Healthcare) for pOPIN-K constructs. Vector-encoded tags were released using either 3C protease for pOPIN-B and pOPIN-K constructs or SENP1 for pOPIN-S constructs. Proteins were further purified using size exclusion chromatography on a HiLoad 16/60 Superdex 75 column (GE Healthcare), concentrated and stored at -80°C.

#### UbiCRest

Ubiquitinated mMHCII or GFP-SteD were purified from Mel JuSo cells stably expressing GFP-SteD (using L243 antibody or GFPTrap beads respectively). After washes, beads were resuspended in dilution buffer (25 mM Tris pH 7.5, 150 mM NaCl) and 12 μL samples (corresponding to 2 x 10^6^ cells) were aliquoted per tube. DUBs were thawed and diluted in buffer (25 mM Tris pH 7.5, 150 mM NaCl, 10 mM DTT) at 5 x working concentration (5 μM USP21, 5 μM vOTU, 5 μM Cezanne, 5 μM OTUB1, 5 μM AMSH, 0.5 μM Otulin). Ubiquitinated substrates (12 μL of GFP-SteD or mMHCII), 1.75 μL 10 x reaction buffer (500 mM Tris pH 7.5, 500 mM NaCl, 50 mM DTT) and 3.5 μL of 5 x DUBs were mixed in Eppendorf tubes at 37^°^ C for 45 min. SDS-PAGE loading buffer was added and samples boiled before SDS PAGE and immunoblotting for ubiquitin using P4D1 antibody.

#### Standard Mass Spectrometry of GFP-SteD

GFP-SteD was obtained from Mel Juso cells stably expressing GFP-SteD using GFP-trap beads. The protein was subjected to an “on-the-beads digestion” procedure in which proteins were reduced with 50 mM TCEP (for 60 min at 60°C), alkylated with 200 mM methyl methanethiosulfonate (45 min at room temperature) and digested overnight with trypsin (sequencing Grade Modified Trypsin - Promega V5111). Peptide mixtures were analyzed by LC-MS-MS/MS (liquid chromatography coupled to tandem mass spectrometry) using Nano-Acquity (Waters) LC system and Orbitrap Velos mass spectrometer (Thermo Electron Corp., San Jose, CA). Prior to the analysis, the peptide mixture was applied to RP-18 precolumn (nanoACQUITY Symmetry® C18 – Waters 186003514) using water containing 0.1% TFA as mobile phase and then transferred to nano-HPLC RP-18 column (nanoACQUITY BEH C18 - Waters 186003545) using an acetonitrile gradient (5% - 35% AcN) in the presence of 0.05% formic acid with the flowrate of 250 nL/min. Column outlet was directly coupled to the ion source of the spectrometer working in the regime of data dependent MS to MS/MS switch. A blank run ensuring lack of cross contamination from previous samples preceded each analysis.

Acquired raw data were processed by Mascot Distiller followed by Mascot Search (Matrix Science, London, UK, on-site license) against NCBInr database (version May, 2016) restricted to bacterial sequences. Search parameters for precursor and product ions mass tolerance were 30 ppm and 0.1 Da, respectively, enzyme specificity: trypsin, missed cleavage sites allowed: 1, fixed methylthio modification of cysteine and variable modification of ubiquitination and methionine oxidation. Peptides with Mascot Score exceeding the threshold value corresponding to < 5% expectation value, calculated by Mascot procedure, and with the Mascot score above 30 were considered to be positively identified.

#### AQUA Mass Spectrometry

Immunoprecipated samples were resolved on 4-12% NuPAGE Bis-Tris gels (Invitrogen) and stained with Instant Blue (Expedeon). Regions of the gel above the mass of the unmodified substrate were separated into two fractions. The light and heavy chain protein bands from the mMHCII immunoprecipitation were avoided. Gel samples were sliced into 1 mm^3^ cubes and subjected to in-gel trypsinization at 37°C for 16 hr. To each trypsinized sample, 2 pmoles of isotopically-labeled ubiquitin standards were added according to (Kirkpatrick et al., 2006). Following lyophilization, the samples were resuspended in 25 μL of reconstitution buffer (7.5% acetonitrile, 0.5% trifluoroacetic acid, 0.01% H_2_O_2_). Methionine-containing peptides were oxidized according to (Phu et al., 2011). 10 μL of each sample was injected onto a Dionex Ultimate 3000 HPLC system and peptides bound to a nanoEase MZ Symmetry trap (C18, 5 μM, 100 Å, 180 μm x 20 cm; Waters, Milford, MA) at a flow rate of 300 nL/min. Prior to ionization, peptides were further separated using an nanoEase MZ HSS T3 column (1.8 μm, 100 Å, 75 μm x 25 cm; Waters). Mass analysis was performed on a Q Exactive (Thermo Scientific) using a parallel reaction-monitoring assay. Skyline software (MacLean et al., 2010) was used to quantify transition ions from the unlabeled and AQUA peptides. Analysis of each gel fraction independently revealed the vast majority of ubiquitin modifications corresponded to 98 kDa or below, and these regions were pooled for subsequent analysis.

#### T Cell Proliferation Assay

MutuDCs that had been exposed to scrambled control gRNA and *Tmem127*^*-/-*^ MutuDC were infected at an MOI of 20:1 in IMDM-treated Petri dishes, cells were centrifuged at 110 *g* for 5 min and incubated at  37°C in 5 % CO_2_ for 30 min. Cells were washed 3 times with PBS and incubated in fresh medium containing gentamicin (100 μg mL^−1^) for 1 h to kill extracellular bacteria. After 1 h, the antibiotic concentration was reduced to 20 μg mL^−1^ and cells were detached using 1mM EDTA inn PBS. Cells (5 x 10^4^) were seeded in 96-well plates and incubated with OVA peptide (ISQAVHAAHAEINEAGR) for 1 h in 5 mM in IMDM containing 10% FCS and 20 μg mL^−1^ gentamicin. After 20 h of infection, cells were transferred to RPMI 1640 medium and incubated with T cells in 96-well plates. T cells expressing OVA-specific T cell receptor (TCR) were isolated from cell suspensions of spleens and lymph nodes of OT-II mice by magnetic sorting of CD4^+^ cells (Miltenyi Biotec) and labeled with CFSE as described previously (Quah and Parish, 2010). CD4^+^ T cells were incubated with DCs at a ratio of 3:1 in a final volume of 200 μL of RPMI 1640 medium containing 20 mg mL^-1^ gentamicin. After 72 h, cells were centrifuged and resuspended in 50 μL FACS buffer containing antibodies and subjected to Flow Cytometry (see above).

### Quantification and Statistical Analysis

Statistical significance was calculated using two-way ANOVA, one-way ANOVA followed by Tukey’s multiple comparison test or one- or two- sample T test correction as indicated in figure legends. All statistical analysis was carried out using GraphPad Prism v8 software.

## Supplemental Information

Document S1. Figures S1–S7 and Table S2

Table S1. Deep Sequencing Results of gRNAs from Sorted Mutants Displaying High mMHCII Surface Levels from Two Independent CRISPR Screens, and from Control (Non-sorted) Cells, Related to Figure 1

Document S2. Article plus Supplemental Information
