# Supplementary material for: The Tumour Suppressor TMEM127 Is a Nedd4-Family E3 Ligase Adaptor Required by Salmonella SteD to Ubiquitinate and Degrade MHC Class II Molecules
Source: Cell Host Microbe. 2020 Jul 8;28(1):54–68.e7. doi: 10.1016/j.chom.2020.04.024 (PMC7342019; doi:10.1016/j.chom.2020.04.024)
Supplement: Document S1. Figures S1–S7 and Table S2 [file mmc1.pdf]

Cell Host & Microbe, Volume *28*

Supplemental Information

The Tumour Suppressor TMEM127 Is a Nedd4-Family E3 Ligase Adaptor Required by *Salmonella* SteD to Ubiquitinate and Degrade MHC Class II Molecules

Eric Alix, Camilla Godlee, Ondrej Cerny, Samkeliso Blundell, Romina Tocci, Sophie Matthews, Mei Liu, Jonathan N. Pruneda, Kirby N. Swatek, David Komander, Tabitha Sleap, and David W. Holden

Figure S1.

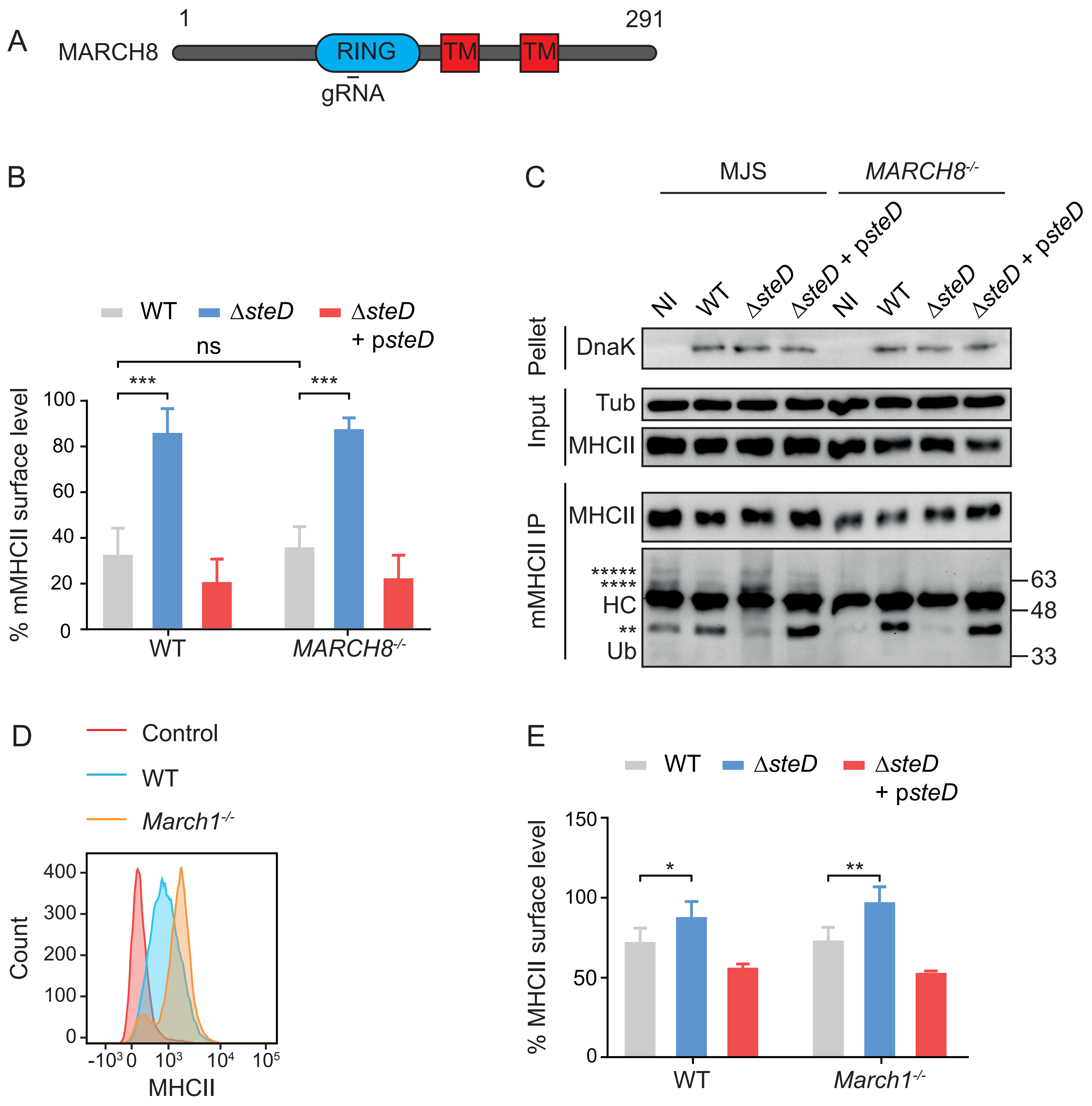
A
MARCH8
1
291
RING
TM
TM
gRNA
B
WT
ΔsteD
ΔsteD + psteD
ns
***
***
% mMHCII surface level
100
80
60
40
20
0
WT
MARCH8-/-
C
MJS
MARCH8-/-
NI
WT
ΔsteD
ΔsteD + psteD
NI
WT
ΔsteD
ΔsteD + psteD
Pellet
DnaK
Input
Tub
MHCII
mMHCII IP
MHCII
*****
****
HC
**
Ub
63
48
33
D
Control
WT
March1-/-
Count
400
300
200
100
0
-10³ 0 10³ 10⁴ 10⁵
MHCII
E
WT
ΔsteD
ΔsteD + psteD
% MHCII surface level
150
100
50
0
*
**
WT
March1-/-

Figure S1. MARCH E3 ligases are not required for SteD-dependent MHCII regulation, Related to Figure 1.

(A) Schematic of MARCH8 indicating the localization of the gRNAs in the knockout used in B. RING – RING finger domain. TM - transmembrane domain.

(B) Quantification of mMHCII surface levels in wild-type (WT) or *MARCH8*$^{-/-}$ Mel Juso cells infected with the indicated *Salmonella* strains. Cells were analysed by flow cytometry 20 h p.i. and amounts of surface mMHCII in infected cells are expressed as a percentage of uninfected cells in the same sample. Data are from 3 independent experiments and show means ± SD. *** $p < 0.001$, ns – non-significant (two-way ANOVA followed by Tukey's multiple comparison test).

(C) Wild-type (WT) or *MARCH8*$^{-/-}$ Mel Juso cells, non-infected (NI) or infected with the indicated *Salmonella* strains were lysed and proteins were immunoprecipitated with L243 antibody. Samples were analysed by immunoblot using anti-DRα (MHCII), anti-tubulin (Tub), anti-ubiquitin (Ub) and anti-DnaK (as a marker for *Salmonella*) antibodies. HC – IgG heavy chain. Protein size markers (kDa) are indicated on right. Ubiquitin blot detects ubiquitinated mMHCII β chain (unmodified β chain – 29 KDa). Bands corresponding to di-, tetra- and penta-ubiquitinated mMHCII are indicated by **, **** and *****.

(D) Representative FACS histograms showing surface levels of total MHCII in wild-type (WT) or *March1*$^{-/-}$ MutuDCs. Control – unlabelled sample. The plots are representative of three independent experiments.

(E) Quantification of total MHCII surface levels in wild-type (WT) or *March1*$^{-/-}$ MutuDCs infected with the indicated *Salmonella* strains. Cells were analysed by flow cytometry 20 h p.i. and amounts of total surface MHCII in infected cells as a percentage of those in uninfected cells from the same sample. Data are from 3 independent experiments and show means ± SD. ** $p < 0.01$, * $p < 0.05$ (Student's T-test).

Figure S2.

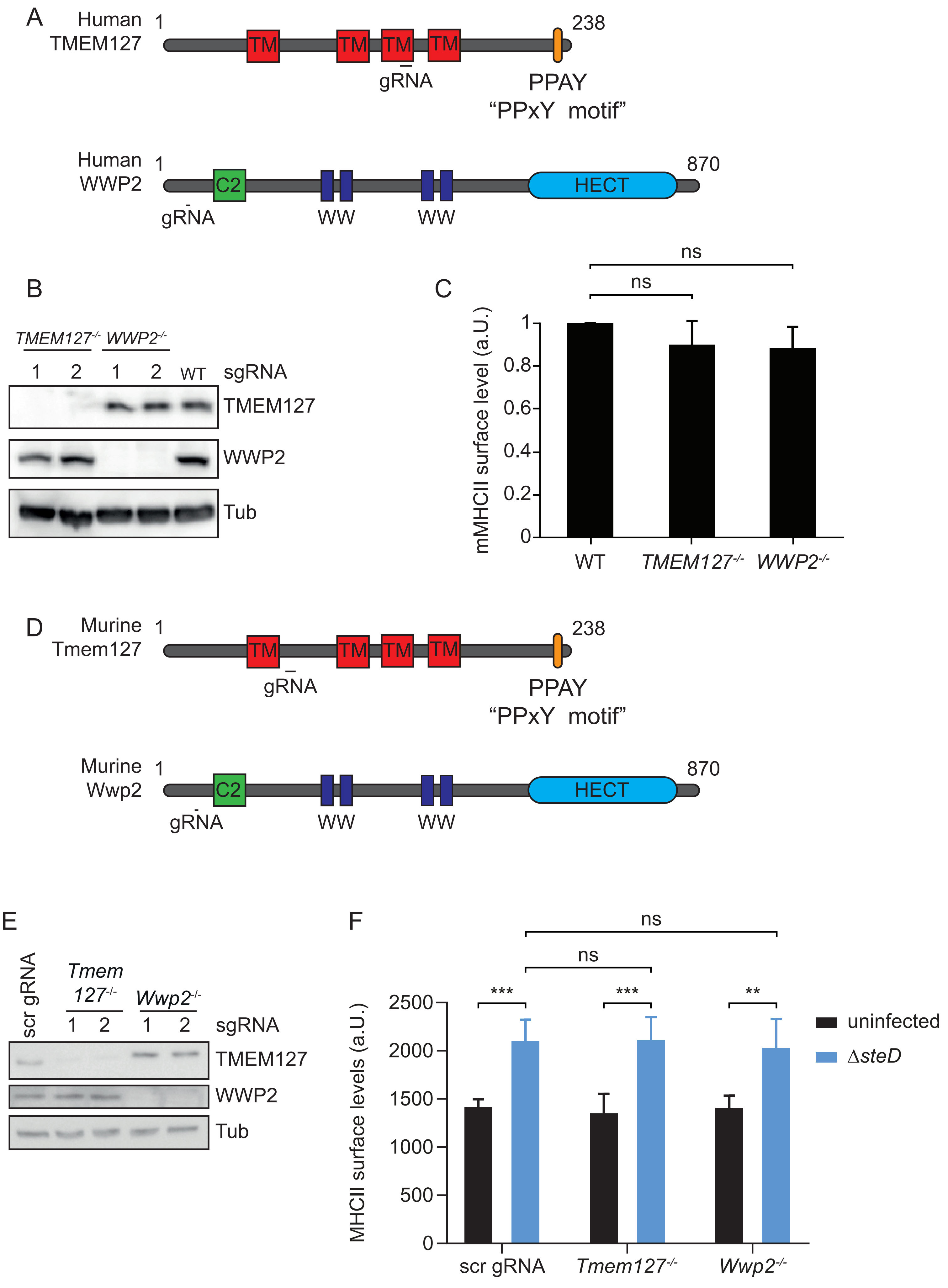
A
Human TMEM127
1
238
TM
TM
TM
TM
gRNA
PPAY
“PPxY motif”
Human WWP2
1
870
C2
HECT
gRNA
WW
WW
B
TMEM127-/-
WWP2-/-
1
2
1
2
WT
sgRNA
TMEM127
WWP2
Tub
C
ns
ns
mMHCII surface level (a.U.)
1
0.8
0.6
0.4
0.2
0
WT
TMEM127-/-
WWP2-/-
D
Murine Tmem127
1
238
TM
TM
TM
TM
gRNA
PPAY
“PPxY motif”
Murine Wwp2
1
870
C2
HECT
gRNA
WW
WW
E
scr gRNA
Tmem 127-/-
Wwp2-/-
1
2
1
2
sgRNA
TMEM127
WWP2
Tub
F
ns
ns
***
***
**
MHCII surface levels (a.U.)
2500
2000
1500
1000
500
0
scr gRNA
Tmem127-/-
Wwp2-/-
uninfected
ΔsteD

Figure S2. Generation of *WWP2*$^{-/-}$ and *TMEM127*$^{-/-}$ Mel Juso cells and *Wwp2*$^{-/-}$ and *Tmem127*$^{-/-}$ MutuDCs, Related to Figures 2 and 3.

(A) Schematic of human TMEM127 and WWP2 showing positions of the gRNA used to make Mel Juso cell knockouts in all further experiments. The PPxY motif and predicted transmembrane domains (TM) are indicated in TMEM127, and the C2 lipid-binding domain, WW protein-interaction domain and HECT E3 ubiquitin ligase domain are shown for WWP2.

(B) Immunoblot analysis of mutant Mel Juso cell lysates showing absence of TMEM127 or WWP2 in two independent knockout clones. Clones 1 were used for subsequent experiments. Blots were probed with antibodies against TMEM127, WWP2 and tubulin (Tub).

(C) Quantification of mMHCII surface levels in wild-type (WT), *TMEM127*$^{-/-}$ or *WWP2*$^{-/-}$ Mel Juso cells. Cells were analysed by flow cytometry and levels of surface mMHCII from 3 independent experiments are indicated with means ± SD. ns – non-significant (one sample T-test).

(D) Schematic of murine Tmem127 and Wwp2 showing positions of the gRNA used to make MutuDC cell knockouts in all further experiments. The PPxY motif and predicted transmembrane domains (TM) are indicated in Tmem127, and the C2 lipid-binding domain, WW protein-interaction domain and HECT E3 ubiquitin ligase domain are shown for Wwp2.

(E) Immunoblot analysis of mutant MutuDC lysates showing absence of Tmem127 or Wwp2 in two independent knockout MutuDC clones. Clones 1 were used for subsequent experiments. Blots were probed with antibodies against TMEM127, WWP2 and tubulin (Tub).

(F) Quantification of total MHCII surface levels in scrambled (scr) gRNA, *Tmem127*$^{-/-}$ or *Wwp2*$^{-/-}$ MutuDC cells. Cells were analysed by flow cytometry and levels of total surface MHCII from 3 independent experiments are indicated with means ± SD. *** $p < 0.001$. ** $p < 0.01$. ns not significant (two-way ANOVA followed by Tukey's multiple comparison test).

Figure S3.

A

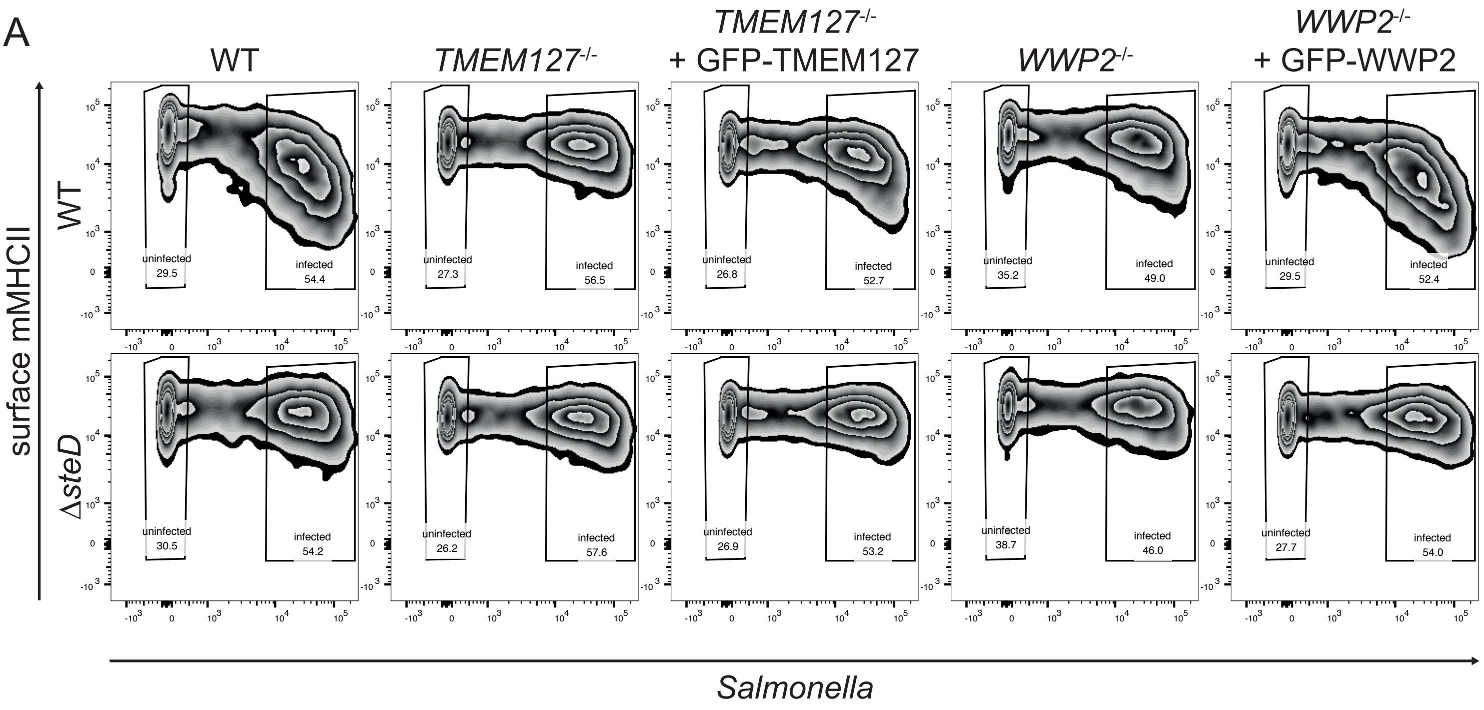
WT
TMEM127-/-
TMEM127-/- + GFP-TMEM127
WWP2-/-
WWP2-/- + GFP-WWP2
surface mMHCII
WT
ΔsteD
Salmonella
uninfected 29.5
infected 54.4
uninfected 27.3
infected 56.5
uninfected 26.8
infected 52.7
uninfected 35.2
infected 49.0
uninfected 29.5
infected 52.4
uninfected 30.5
infected 54.2
uninfected 26.2
infected 57.6
uninfected 26.9
infected 53.2
uninfected 38.7
infected 46.0
uninfected 27.7
infected 54.0

B

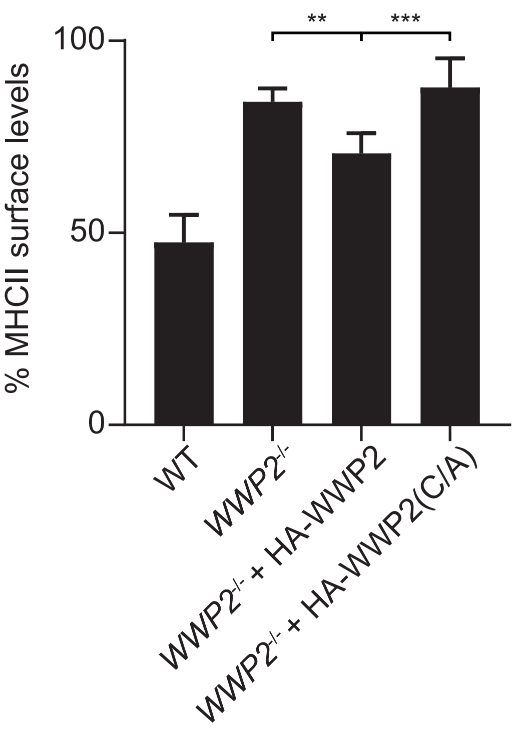
% MHCII surface levels
100
50
0
**
***
WT
WWP2-/-
WWP2-/- + HA-WWP2
WWP2-/- + HA-WWP2(C/A)

Figure S3. Complementation of *WWP2*$^{-/-}$ and *TMEM127*$^{-/-}$ Mel Juso cells, Related to Figure 2.

(A) Representative flow cytometry plots showing surface levels of mMHCII in infected cells compared to uninfected cells. Wild-type (WT), mutant and complemented Mel JuSo cells were infected with wild-type (WT) or *steD* mutant mCherry-expressing *Salmonella.* Numbers shown are percentage of total cells in each gate. Y axis: Surface mMHCII L243 antibody; X axis: mCherry levels.

(B) Quantification of mMHCII surface levels in wild-type (WT) or *WWP2*$^{-/-}$ Mel JuSo cells expressing HA-tagged WWP2 or a catalytic dead point mutant of WWP2 (C/A) infected with mCherry-expressing wild-type *Salmonella*. Cells were analysed by flow cytometry and amounts of surface mMHCII in infected cells are expressed as a percentage of uninfected cells in the same sample. Data are from 3 independent experiments and show means ± SD. ** $p < 0.01$, ***$p < 0.001$ (one-way ANOVA followed by Tukey's multiple comparison test).

Figure S4.

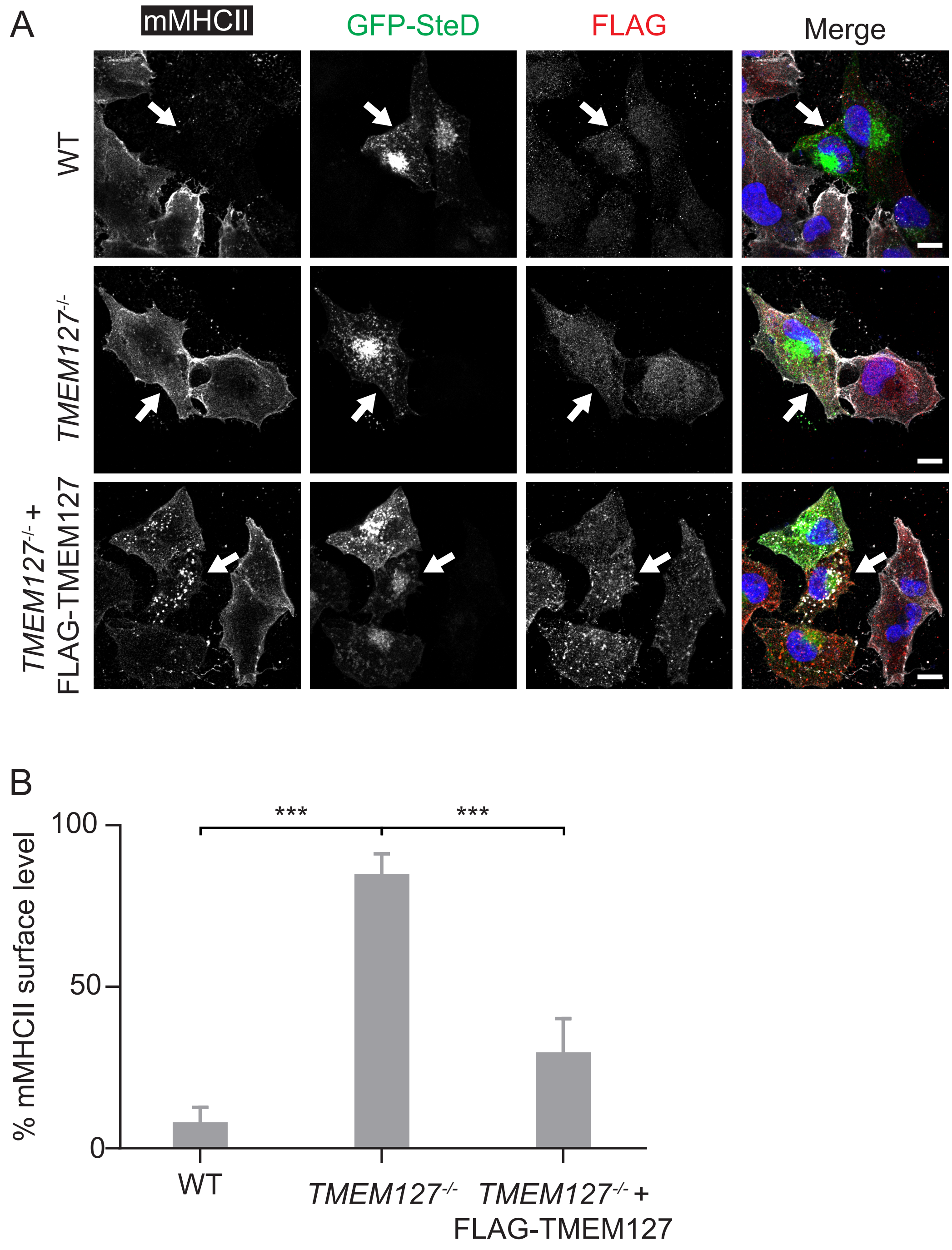
A
mMHCII
GFP-SteD
FLAG
Merge
WT
TMEM127-/-
TMEM127-/- +
FLAG-TMEM127
B
% mMHCII surface level
100
50
0
***
***
WT
TMEM127-/-
TMEM127-/- +
FLAG-TMEM127

Figure S4. FLAG-TMEM127 complements requirement of TMEM127 for SteD-dependent reduction of surface mMHCII, Related to Figures 4 and 5.

(A) Representative confocal immunofluorescence microscopy images of wild-type (WT) or *TMEM127*$^{-/-}$ Mel JuSo cells expressing FLAG-TMEM127 (red) and GFP-SteD (green). Cells were fixed and labeled for surface mMHCII (L243 antibody, white). Arrows show transfected cells. Scale bar - 10 µm.

(B) Quantification of total mMHCII surface levels in wild-type (WT), *TMEM127*$^{-/-}$ or *TMEM127*$^{-/-}$ cells stably expressing FLAG-TMEM127, transiently expressing GFP-SteD. Cells were analysed by flow cytometry and amounts of surface mMHCII in GFP-positive cells are expressed as a percentage of GFP-negative cells in the same sample. Data are from 3 independent experiments and show means ± SD. *** $p < 0.001$ (one-way ANOVA followed by Tukey's multiple comparison test).

Figure S5.

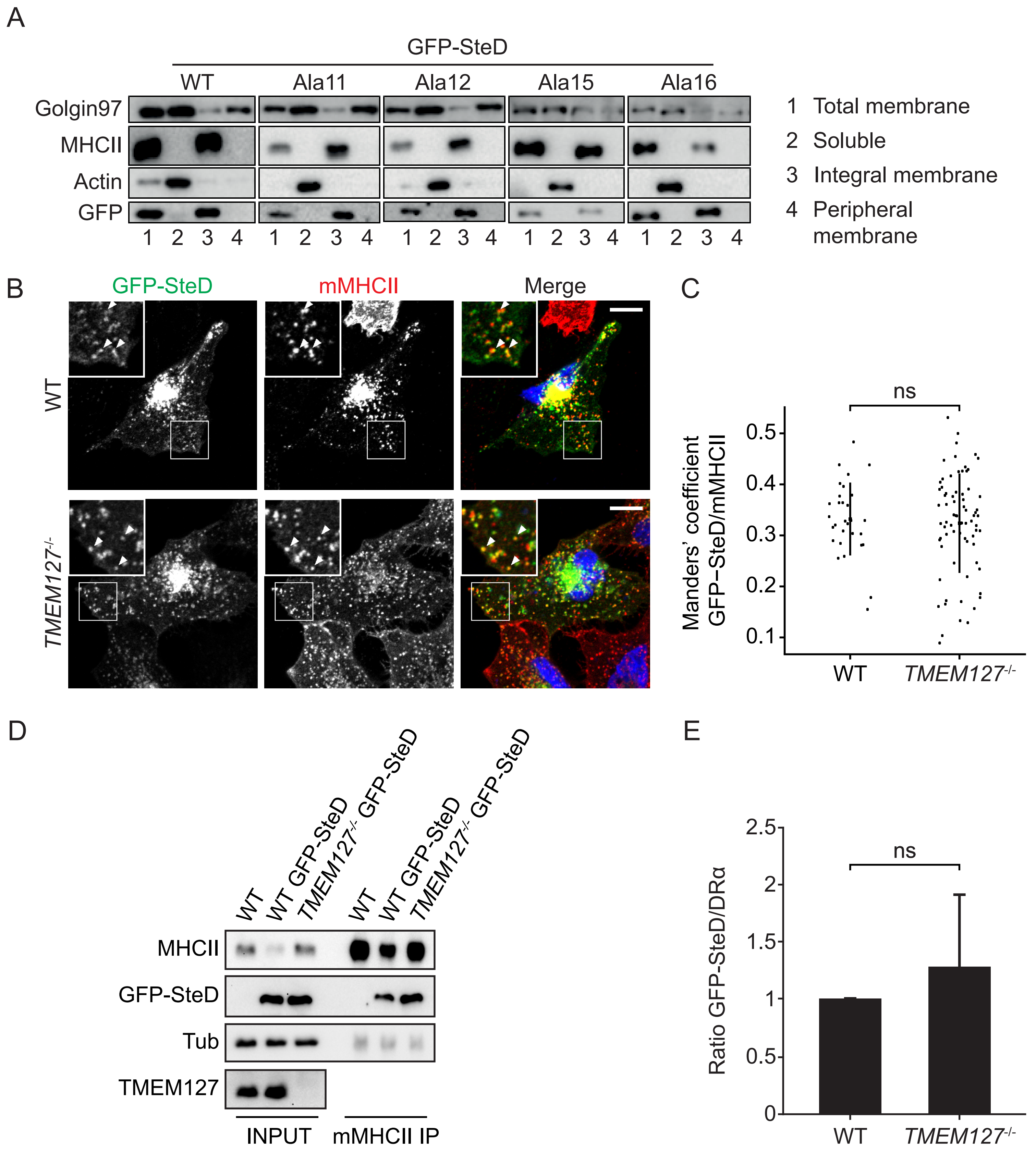
A
GFP-SteD
WT
Ala11
Ala12
Ala15
Ala16
Golgin97
MHCII
Actin
GFP
1 2 3 4 1 2 3 4 1 2 3 4 1 2 3 4 1 2 3 4
1 Total membrane
2 Soluble
3 Integral membrane
4 Peripheral membrane
B
GFP-SteD
mMHCII
Merge
WT
TMEM127-/-
C
ns
Manders' coefficient GFP-SteD/mMHCII
0.5
0.4
0.3
0.2
0.1
WT
TMEM127-/-
D
WT
WT GFP-SteD
TMEM127-/- GFP-SteD
WT
WT GFP-SteD
TMEM127-/- GFP-SteD
MHCII
GFP-SteD
Tub
TMEM127
INPUT
mMHCII IP
E
ns
Ratio GFP-SteD/DRα
2.5
2
1.5
1
0.5
0
WT
TMEM127-/-

Figure S5. Analysis of the interactions between SteD, TMEM127 and MHCII, Related to Figure 5.

(A) Mel Juso cells expressing GFP-SteD (WT) or four alanine (Ala) mutants (as shown in Fig. 5C) were lysed and the total membrane fraction was separated from soluble proteins by centrifugation. Peripherally-associated proteins were separated from integral membrane proteins by washing the membrane fraction with 2.5 M urea followed by centrifugation. The different fractions were analysed by immunoblot using anti-Golgin97, anti-DRα (MHCII), anti-Actin and anti-GFP antibodies.

(B) Representative confocal immunofluorescence microscopy images of wild-type (WT) or *TMEM127*$^{-/-}$ Mel Juso cells expressing GFP-SteD (green). Cells were fixed and labeled for total mMHCII (L243 antibody, red) and stained with DAPI (blue). Magnified boxed areas show vesicular colocalization of the two proteins (arrowheads). Scale bar - 10 μm.

(C) Mander's overlap coefficient of the fraction of GFP-SteD positive pixels that colocalise with mMHCII positive pixels in wild-type (WT) or *TMEM127*$^{-/-}$ Mel JuSo cells. Data are representative of three independent experiments. Each dot represents the value for one cell. Error bars show mean ± SD. ns – not significant (Student's T-test).

(D) Wild-type (WT) or *TMEM127*$^{-/-}$ Mel JuSo cells expressing GFP-SteD (where indicated) were lysed and proteins were immunoprecipitated with L243 antibody. Samples were analysed by immunoblot using anti-DRα (MHCII), anti-GFP, anti-tubulin (Tub) and anti-TMEM127 antibodies.

(E) Quantification of intensity of GFP-SteD signal in immunoprecipitates in (D) relative to immunoprecipitated MHCII (DRα) in wild-type (WT) or *TMEM127*$^{-/-}$ Mel JuSo cells. Data show means ± SD from 3 independent experiments. ns – not significant (Student's T-test).

Figure S6.

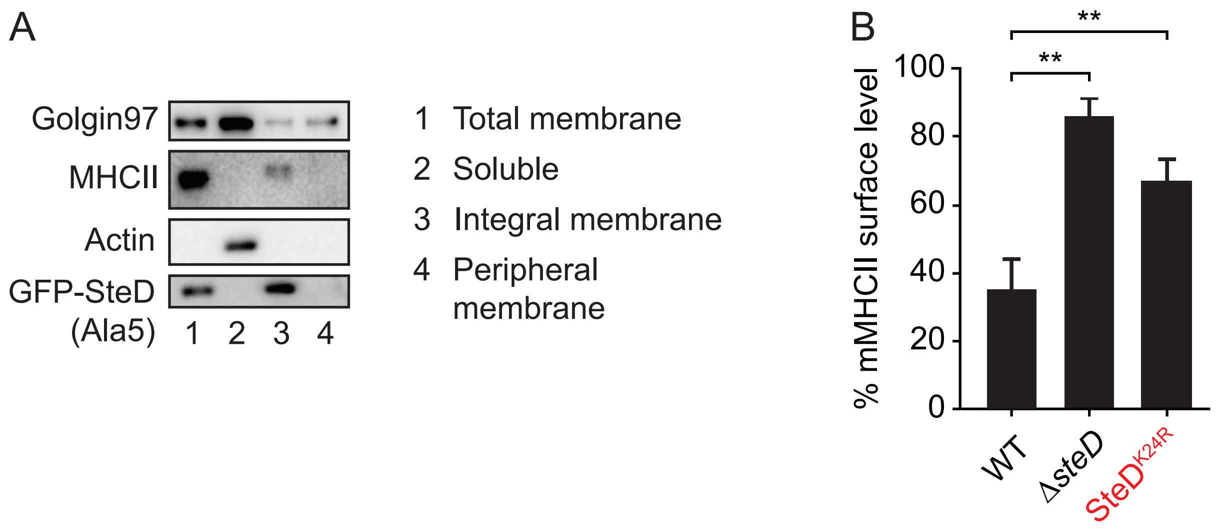
A
Golgin97
MHCII
Actin
GFP-SteD
(Ala5)
1 2 3 4
1 Total membrane
2 Soluble
3 Integral membrane
4 Peripheral membrane
B
% mMHCII surface level
100
80
60
40
20
0
**
**
WT
ΔsteD
SteDK24R

Figure S6. Analysis of the K24R SteD mutant, Related to Figure 6.

(A) Mel Juso cells stably expressing the Ala5 mutant of GFP-SteD (as shown in Fig. 5C) were lysed and the total membrane fraction was separated from soluble proteins by centrifugation. Peripherally-associated proteins were separated from integral membrane proteins by washing the membrane fraction with 2.5 M urea followed by centrifugation. The different fractions were analysed by immunoblot using anti-Golgin97, anti-DRα (MHCII), anti-Actin and anti-GFP antibodies.

(B) Quantification of mMHCII surface levels in Mel Juso cells infected with the indicated *Salmonella* strains for 20 h. $SteD^{K24R}$ expressed from the chromosome is indicated in red. Cells were analysed by flow cytometry 20 h p.i. and amounts of surface mMHCII are expressed as a percentage of uninfected cells from the same sample. Data are from 3 independent experiments and show means ± SD. ** $p < 0.01$ (one-way ANOVA followed by Tukey's multiple comparison test).

Figure S7

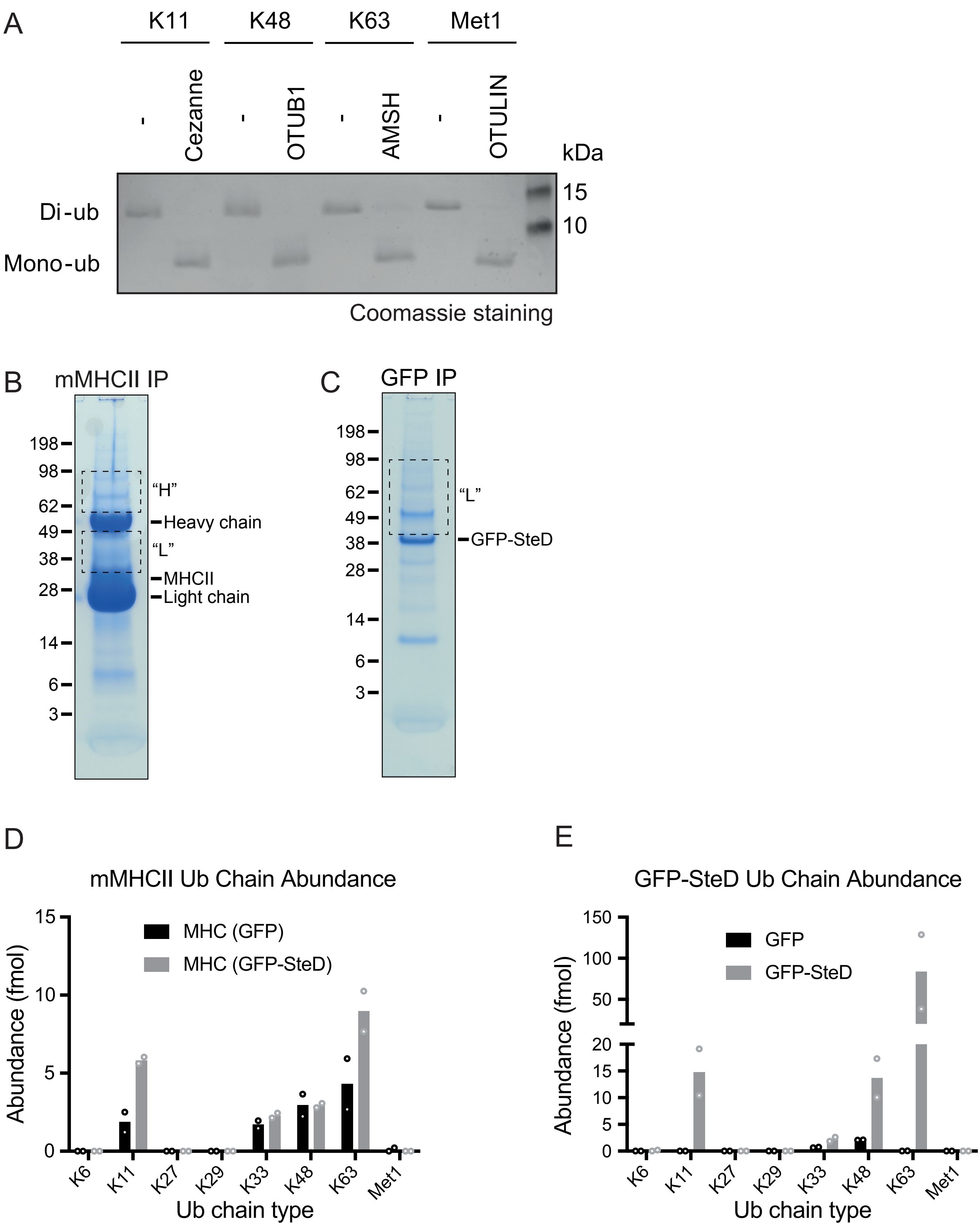
A
K11
K48
K63
Met1
-
Cezanne
-
OTUB1
-
AMSH
-
OTULIN
kDa
15
10
Di-ub
Mono-ub
Coomassie staining
B
mMHCII IP
198
98
62
49
38
28
14
6
3
"H"
Heavy chain
"L"
MHCII
Light chain
C
GFP IP
198
98
62
49
38
28
14
6
3
"L"
GFP-SteD
D
mMHCII Ub Chain Abundance
Abundance (fmol)
15
10
5
0
MHC (GFP)
MHC (GFP-SteD)
K6
K11
K27
K29
K33
K48
K63
Met1
Ub chain type
E
GFP-SteD Ub Chain Abundance
Abundance (fmol)
150
100
50
20
15
10
5
0
GFP
GFP-SteD
K6
K11
K27
K29
K33
K48
K63
Met1
Ub chain type

Figure S7. SteD and mMHCII are ubiquitinated predominantly with K63-linked chains, Related to Figure 7.

(A) Enzymes used for UbiCREST assay are functional. Di-ubiquitin chains of indicated linkage types were incubated with the corresponding specific deubiquitinase or buffer only (-). Ubiquitin was separated by SDS-PAGE and detected by Coomassie Blue staining. Protein size markers (kDa) are indicated on right.

(B) Mel Juso cells stably expressing GFP-SteD were lysed and mMHCII was immunoprecipitated using L243 antibody. Samples were separated by SDS-PAGE and stained with Coomassie Blue. Fractions cut from gels and analysed by AQUA mass spectrometry are labelled as "L" and "H".

(C) Mel Juso cells expressing GFP-SteD were lysed and GFP-SteD was precipitated using GFP-trap beads. Samples were separated by SDS-PAGE and stained with Coomassie Blue. Fraction cut from gel and analysed by AQUA mass spectrometry is labelled as "L".

(D) Abundance of each type of ubiquitin linkage associated with purified mMHCII in the presence or absence of SteD by AQUA mass spectrometry (Fractions L + H).

(E) Abundance of each type of ubiquitin linkage associated with purified GFP-SteD compared to GFP by AQUA mass spectrometry (Fraction L).

Table S2. Primer sequences. Related to STAR Methods

Use	Name	Sequence (5'-3')
amplification sgRNAs	NGS-Lib-Fwd-1	AATGATACGGCGACCACCGAGATCTA<br>CACTCTTTCCCTACACGACGCTCTTCC<br>GATCTTAAGTAGAGGCTTTATATATCT<br>TGTGGAAAGGACGAAACACC
	NGS-Lib-Fwd-2	AATGATACGGCGACCACCGAGATCTA<br>CACTCTTTCCCTACACGACGCTCTTCC<br>GATCTATCATGCTTAGCTTTATATATC<br>TTGTGGAAAGGACGAAACACC
	NGS-Lib-Fwd-3	AATGATACGGCGACCACCGAGATCTA<br>CACTCTTTCCCTACACGACGCTCTTCC<br>GATCTGATGCACATCTGCTTTATATAT<br>CTTGTGGAAAGGACGAAACACC
	NGS-Lib-Fwd-4	AATGATACGGCGACCACCGAGATCTA<br>CACTCTTTCCCTACACGACGCTCTTCC<br>GATCTCGATTGCTCGACGCTTTATATA<br>TCTTGTGGAAAGGACGAAACACC
	NGS-Lib-Fwd-5	AATGATACGGCGACCACCGAGATCTA<br>CACTCTTTCCCTACACGACGCTCTTCC<br>GATCTTCGATAGCAATTCGCTTTATAT<br>ATCTTGTGGAAAGGACGAAACACC
	NGS-Lib-Fwd-6	AATGATACGGCGACCACCGAGATCTA<br>CACTCTTTCCCTACACGACGCTCTTCC<br>GATCTATCGATAGTTGCTTGCTTTATA<br>TATCTTGTGGAAAGGACGAAACACC
	NGS-Lib-Fwd-7	AATGATACGGCGACCACCGAGATCTA<br>CACTCTTTCCCTACACGACGCTCTTCC<br>GATCTGATCGATCCAGTTAGGCTTTAT<br>ATATCTTGTGGAAAGGACGAAACACC
	NGS-Lib-Fwd-8	AATGATACGGCGACCACCGAGATCTA<br>CACTCTTTCCCTACACGACGCTCTTCC<br>GATCTCGATCGATTTGAGCCTGCTTTA<br>TATATCTTGTGGAAAGGACGAAACAC C
	NGS-Lib-Fwd-9	AATGATACGGCGACCACCGAGATCTA<br>CACTCTTTCCCTACACGACGCTCTTCC<br>GATCTACGATCGATACACGATCGCTTT<br>ATATATCTTGTGGAAAGGACGAAACA CC
	NGS-Lib-Fwd-10	AATGATACGGCGACCACCGAGATCTA<br>CACTCTTTCCCTACACGACGCTCTTCC<br>GATCTTACGATCGATGGTCCAGAGCTT<br>TATATATCTTGTGGAAAGGACGAAAC ACC
	NGS-Lib-KO-Rev- 1	CAAGCAGAAGACGGCATACGAGATTC<br>GCCTTGGTGACTGGAGTTCAGACGTG<br>TGCTCTTCCGATCTCCGACTCGGTGCC<br>ACTTTTTCAA
	NGS-Lib-KO-Rev- 2	CAAGCAGAAGACGGCATACGAGATAT<br>AGCGTCGTGACTGGAGTTCAGACGTG<br>TGCTCTTCCGATCTCCGACTCGGTGCC<br>ACTTTTTCAA
	NGS-Lib-KO-Rev- 3	CAAGCAGAAGACGGCATACGAGATGA<br>AGAAGTGTGACTGGAGTTCAGACGTG<br>TGCTCTTCCGATCTCCGACTCGGTGCC<br>ACTTTTTCAA

	NGS-Lib-KO-Rev- 4	CAAGCAGAAGACGGCATACGAGATAT TCTAGGGTGACTGGAGTTCAGACGTG TGCTCTTCCGATCTCCGACTCGGTGCC ACTTTTTCAA
	NGS-Lib-KO-Rev- 5	CAAGCAGAAGACGGCATACGAGATCG TTACCAGTGACTGGAGTTCAGACGTG TGCTCTTCCGATCTCCGACTCGGTGCC ACTTTTTCAA
SteD K24R construction	ovK24R-F	tgacgaacgaccggtagctgaaatc
	ovK24R-R	ctaccggtcgttcgtcattaccccg
	SteD-Hind-F	tgcaacaagcttgatatcgaattctgataacg
	SteD-Sac-R	tgcaacgagctccaccgcggtcacgtataa
sgRNA MARCH8	MARCH8-1	CACCGGGCAGGCCTGGTGCACGAAG
	MARCH8-2	AAACCTTCGTGCACCAGGCCTGCCC
	MARCH8-3	CACCGAGTGGCAGGGGGTGATCAGG
	MARCH8-4	AAACCCTGATCACCCCCTGCCACTC
	MARCH8-5	CACCGAGGCCTGGTGCACGAAGTGG
	MARCH8-6	AAACCCACTTCGTGCACCAGGCCTC
sequence lentiguide-puro	hGata4-rev	ATTGTGGATGAATACTGCC
K24R chromosomal mutation	pGP704_SteD_R_SacI	aaccggGAGCTCggttatctataataaatgagc
	pGP704_SteD_F_SalI	aaccggGTCGACcgaacatacgtcagttatcag
	pWRG100_dsteD_F	tctcataaacataaacaggcatgtgcatgaagaggtttat cgccttacgccccgccctgc
	pWRG100_dsteD_R	ggaattcgtcttttccctatgacttgctgtgtttgctcat ctagactatattaccctgtt
cloning HA-SteD into M6P	M6P_Nco_SteD_F	AAGCCATGGGAGGAtatccgtatgatgtgccggactatgcgtat ccgtatgatgttcctgattatgctGGAatgaatgtcacttcaggcgtg
	M6P_Not_SteD_R	AACGCGGCCGCTTAtggccaggctggccgggttctg
sgRNA MJS lentiguide	TMEM127_1_F	CACCGCTGCAGTGTGCCACCGTCAT
	TMEM127_1_R	AAACATGACGGTGGCACACTGCAGC
	TMEM127_2_F	CACCGGTGCACATAGCCCAACACGT
	TMEM127_2_R	AAACACGTGTTGGGCTATGTGCACC
	WWP2_1_F	CACCGACCTCGAATTAACTCCTACG
	WWP2_1_R	AAACCGTAGGAGTTAATTCGAGGTC
	WWP2_2_F	CACCGAGGTTGACGATTATGCACCT
	WWP2_2_R	AAACAGGTGCATAATCGTCAACCTC
sequencing gDNA KOs	gWWP2_F	AAAGAGCCGGAACATCTGCCACAGG
	gWWP2_R	CAGGGAATCTGTACCTCCCACAACC
	gTMEM127_F1	GTCAAGAGAGCTCCTCCTTGCAGAG
	gTMEM127_R1	CCAGCTCCTGCCACCAGGTAGAAGC
	gTMEM127_F2	GAGCGTAGCCTGGCCTCGGCCCTGC
	gTMEM127_R2	TGGGCATGAACACCAGGCAGTTATG
	TMEM127-BsmBI_NcoI_Flag-F	aaccgtctcacatggccgattacaaggatgacgacgataaggctggaatgtac gcccccggagg
	TMEM127-BsmBI_NotI-R	aaccgtctcaggccgcttagggtgtgtaagcaggg
	TMEM127-BsmBI_PciI_F	aaccgtctcacatgtccatgtacgcccccggagg
	TMEM127_PY_BsmBI_NotI-R	aaccgtctcaggccgc ttagggtgtggcagcaggggggtggctggaa

	WWP2-BsmBI_NcoI_Flag-F	aaccgtctcacatggccgattacaaggatgacgacgataaggctggaatggc atctgccagctcta
	WWP2-BsmBI_NotI-R	aaccgtctcaggccgcttactcctgtccaaagccc
	WWP2-BsmBI_PciI_F	aaccgtctcacatgtccatggcatctgccagctcta
sgRNA mouse lentiguide	Mouse_WWP2_sgRNA_1F	CACCGCCCAGCGAGACCAAGAAGAC
	Mouse_WWP2_sgRNA_1R	AAACGTCTTCTTGGTCTCGCTGGGC
	Mouse_WWP2_sgRNA_2F	CACCGACTGCTTTGGTGGCAGATCC
	Mouse_WWP2_sgRNA_2R	AAACGGATCTGCCACCAAAGCAGTC
	Mouse_TMEM127_sgRNA_1F	CACCGTGGGAAGAGCGCTGCTTCCC
	Mouse_TMEM127_sgRNA_1R	AAACGGGAAGCAGCGCTCTTCCCAC
	Mouse_TMEM127_sgRNA_2F	CACCGCGTGCTGGGCTATGTAAACC
	Mouse_TMEM127_sgRNA_2R	AAACGGTTTACATAGCCCAGCACGC
	Mouse_NonTargeting_control_F	CACCGGCGAGGTATTCGGCTCCGCG
	Mouse_NonTargeting_control_R	AAACCGCGGAGCCGAATACCTCGCC
